# Restricted maximum-likelihood method for learning latent variance components in gene expression data with known and unknown confounders

**DOI:** 10.1093/g3journal/jkab410

**Published:** 2021-12-01

**Authors:** Muhammad Ammar Malik, Tom Michoel

**Affiliations:** Computational Biology Unit, Department of Informatics, University of Bergen, Bergen 5020, Norway

**Keywords:** gene expression, random effects model, latent factors, eQTLs

## Abstract

Random effects models are popular statistical models for detecting and correcting spurious sample correlations due to hidden confounders in genome-wide gene expression data. In applications where some confounding factors are known, estimating simultaneously the contribution of known and latent variance components in random effects models is a challenge that has so far relied on numerical gradient-based optimizers to maximize the likelihood function. This is unsatisfactory because the resulting solution is poorly characterized and the efficiency of the method may be suboptimal. Here, we prove analytically that maximum-likelihood latent variables can always be chosen orthogonal to the known confounding factors, in other words, that maximum-likelihood latent variables explain sample covariances not already explained by known factors. Based on this result, we propose a restricted maximum-likelihood (REML) method that estimates the latent variables by maximizing the likelihood on the restricted subspace orthogonal to the known confounding factors and show that this reduces to probabilistic principal component analysis on that subspace. The method then estimates the variance–covariance parameters by maximizing the remaining terms in the likelihood function given the latent variables, using a newly derived analytic solution for this problem. Compared to gradient-based optimizers, our method attains greater or equal likelihood values, can be computed using standard matrix operations, results in latent factors that do not overlap with any known factors, and has a runtime reduced by several orders of magnitude. Hence, the REML method facilitates the application of random effects modeling strategies for learning latent variance components to much larger gene expression datasets than possible with current methods.

## Introduction

Following the success of genome-wide association studies (GWAS) in mapping the genetic architecture of complex traits and diseases in human and model organisms (Mackay *et al.* 2009; Hindorff *et al.* 2009; Manolio 2013), there is now a great interest in complementing these studies with molecular data to understand how genetic variation affects epigenetic and gene expression states (Albert and Kruglyak 2015; Franzén *et al.* 2016; GTEx Consortium 2017). In GWAS, it is well-known that population structure or cryptic relatedness among individuals may lead to confounding that can alter significantly the outcome of the study (Astle and Balding 2009). When dealing with molecular data, this is further exacerbated by the often unknown technical or environmental influences on the data generating process. This problem is not confined to population-based studies—in single-cell analyses of gene expression, hidden subpopulations of cells and an even greater technical variability cause significant expression heterogeneity that needs to be accounted for (Buettner *et al.* 2015).

In GWAS, linear mixed models have been hugely successful in dealing with confounding due to population structure (Yu *et al.* 2006; Astle and Balding 2009; Kang *et al.* 2010; Lippert *et al.* 2011; Zhou and Stephens 2012). In these models, it is assumed that an individual’s trait value is a linear function of fixed and random effects, where the random effects are normally distributed with a covariance matrix determined by the genetic similarities between individuals, hence accounting for confounding in the trait data. Random effect models have also become popular in the correction for hidden confounders in gene expression data (Kang *et al.* 2008; Listgarten *et al.* 2010; Fusi *et al.* 2012), generally outperforming approaches based on principal component analysis (PCA), the singular value decomposition (SVD), or other hidden factor models (Leek and Storey 2007; Stegle *et al.* 2010, 2012). In this context, estimating the latent factors and the sample-to-sample correlations they induce on the observed high-dimensional data is the critical problem to solve.

If it is assumed that the observed correlations between samples are entirely due to latent factors, it can be shown that the resulting random effects model is equivalent to probabilistic PCA, which can be solved analytically in terms of the dominant eigenvectors of the sample covariance matrix (Tipping and Bishop 1999; Lawrence 2005). However, in most applications, some confounding factors are known in advance (*e.g.*, batch effects, genetic factors in population-based studies, or cell-cycle stage in single-cell studies), and the challenge is to estimate simultaneously the contribution of the known as well as the latent factors. This has so far relied on the use of numerical gradient-based quasi-Newton optimizers to maximize the likelihood function (Fusi *et al.* 2012; Buettner *et al.* 2015). This is unsatisfactory because the resulting solution is poorly characterized, the relation between the known and latent factors is obscured, and due to the high-dimensionality of the problem, “limited memory” optimizers have to be employed whose theoretical convergence guarantees are somewhat weak (Liu and Nocedal 1989; Lin *et al.* 2017).

Intuitively, latent variables should explain sample covariances not already explained by known confounding factors. Here, we demonstrate analytically that this intuition is correct: latent variables can always be chosen orthogonal to the known factors without reducing the likelihood or variance explained by the model. Based on this result, we propose a method that is conceptually analogous to estimating fixed and random effects in linear mixed models using the restricted maximum-likelihood (REML) method, where the variance parameters of the random effects are estimated on the restricted subspace orthogonal to the maximum-likelihood estimates of the fixed effects (Gumedze and Dunne 2011). Our method, called Lvreml, similarly estimates the latent variables by maximizing the likelihood on the restricted subspace orthogonal to the known factors, and we show that this reduces to probabilistic PCA on that subspace. It then estimates the variance–covariance parameters by maximizing the remaining terms in the likelihood function given the latent variables, using a newly derived analytic solution for this problem. Similarly to the REML method for conventional linear mixed models, the Lvreml solution is not guaranteed to maximize the total likelihood function. However, we prove analytically that for any given number *p* of latent variables, the Lvreml solution attains minimal unexplained variance among all possible choices of *p* latent variables, arguably a more intuitive and easier to understand criterion.

The inference of latent variables that explain observed sample covariances in gene expression data is usually pursued for two reasons. First, the latent variables, together with the known confounders, are used to construct a sample-to-sample covariance matrix that is used for the downstream estimation of variance parameters for individual genes and improved identification of trans-eQTL associations (Fusi *et al.* 2012; Stegle *et al.* 2012). Second, the latent variables are used directly as “endophenotypes” that are given a biological interpretation and whose genetic architecture is of stand-alone interest (Parts *et al.* 2011; Stegle *et al.* 2012). This study contributes to both objectives. First, we show that the covariance matrix inferred by Lvreml is *identical* to the one inferred by gradient-based optimizers, while computational runtime is reduced by orders of magnitude (*e.g.*, a 10^4^-fold speed-up on gene expression data from 600 samples). Second, latent variables inferred by Lvreml by design do not overlap with already known covariates and thus represent *new* aggregate expression phenotypes of potential interest. In contrast, we show that existing methods infer latent variables that overlap significantly with the known covariates (cosine similarities of up to 30%) and thus represent partially redundant expression phenotypes.

## Materials and methods

### Mathematical methods

All model equations, mathematical results, and detailed proofs are described in a separate Supplementary material document.

### Data

We used publicly available genotype and RNA sequencing data from 1012 segregants from a cross between two yeast strains (Albert *et al.* 2018), consisting of gene expression levels for 5720 genes and (binary) genotype values for 42,052 SNPs. Following Albert *et al.* (2018), we removed batch and optical density effects from the expression data using categorical regression. The expression residuals were centered such that each sample had mean zero to form the input matrix **Y** to the model (cf. SupplementarySection S2). L2-normalized genotype PCs were computed using the SVD of the genotype data matrix with centered (mean zero) samples and used to form input matrices **Z** to the model (cf. SupplementarySection S2). Data preprocessing scripts are available at https://github.com/michoel-lab/lvreml.

### Lvreml analyses

The Lvreml software, as well as a script that details the Lvreml analyses of the yeast data, is available at https://github.com/michoel-lab/lvreml.

### Panama analyses

We obtained the Panama software from the Limix package available at https://github.com/limix/limix-legacy.

The following settings were used to ensure that exactly the same normalized data were used by both methods: (1) For parameter **Y**, the same gene expression matrix, with each sample normalized to have zero mean, was used as input for Lvreml, setting the **standardize** parameter to false. (2) The parameter **Ks** requires a list of covariance matrices for each known factor. Therefore, for each column *z_i_* of the matrix **Z** used by Lvreml, we generated a covariance matrices $Ks_{i}=z_{i}z_{i}^{T}$. The **use Kpop** parameter, which is used to supply a population structure covariance matrix to Panama in addition to the known covariates, was set to false.

To be able to calculate the log-likelihoods and extract other relevant information from the Panama results, we made the following modifications to the Panama code: (1) The covariance matrices returned by Panama are by default normalized by dividing the elements of the matrix by the mean of its diagonal elements. To make these covariance matrices comparable to Lvreml, this normalization was omitted by commenting out the lines in the original Panama code where this normalization was being performed. (2) Panama does not return the variance explained by the known confounders unless the **use Kpop** parameter is set to true. Therefore, the code was modified so that it would still return the variance explained by the known confounders. (3) The **K** matrix returned by Panama does not include the effect of the noise parameter $\sigma^{2}$. Therefore, the code was modified to return the $\sigma^{2}1$ matrix, which was then added to the returned **K**, *i.e.*, $K_{\mathrm{new}}=K+\sigma^{2}1$, to be able to use eq. (2) to compute the log-likelihood. The modified code is available as a fork of the Limix package at https://github.com/michoel-lab/limix-legacy

## Results

### REML solution for a random effects model with known and latent variance components

Our model to infer latent variance components in a gene expression data matrix is the same model that was popularized in the Panama software (Fusi *et al.* 2012) and scLVM software (Buettner *et al.* 2015), where a linear relationship is assumed between expression levels and the known and latent factors, with random noise added (SupplementarySection S2). In matrix notation, the model can be written as

(1) Y=ZV+XW+ϵ,

where $Y\in {\mathbb{R}}^{n\times m}$ is a matrix of gene expression data for *m* genes in *n* samples, and $Z\in {\mathbb{R}}^{n\times d}$$X\in {\mathbb{R}}^{n\times p}$ are matrices of values for *d* known and *p* latent confounders in the same *n* samples. The columns *v_i_* and *w_i_* of the random matrices $V\in {\mathbb{R}}^{d\times m}$ and $W\in {\mathbb{R}}^{p\times m}$ are the effects of the known and latent confounders, respectively, on the expression level of gene *i* and are assumed to be jointly normally distributed:

$$p\left(\left[\begin{array}{c} v_{i}\\ w_{i} \end{array}\right]\right)=N\left(0,\left[\begin{array}{cc} B & D\\ D^{T} & A \end{array}\right]\right)$$

where $B\in {\mathbb{R}}^{d\times d},\;A\in {\mathbb{R}}^{p\times p}$, and $D\in {\mathbb{R}}^{d\times p}$ are the covariances of the known–known, latent–latent, and known–latent confounder effects, respectively. Lastly, $\epsilon \in {\mathbb{R}}^{n\times m}$ is a matrix of independent samples of a Gaussian distribution with mean zero and variance $\sigma^{2}$, independent of the confounding effects.

Previously, this model was considered with independent random effects (**B** and **A** diagonal and D=0; Fusi *et al.* 2012; Buettner *et al.* 2015). As presented here, the model is more general and accounts for possible lack of independence between the effects of the known covariates. Furthermore, allowing the effects of the known and latent factors to be dependent (D≠0) is precisely what will allow the latent variables to be orthogonal to the known confounders (SupplementarySection S6). An equivalent model with D=0 can be considered but requires nonorthogonal latent variables to explain part of the sample covariance matrix, resulting in a mathematically less tractable framework. Finally, it remains the case that we can always choose **A** to be diagonal, because the latent factors have an inherent rotational symmetry that allows any non-diagonal model to be converted to an equivalent diagonal model (SupplementarySection S5). By definition, the known covariates correspond to measured or “natural” variables, and hence, they have no such rotational symmetry.

Using standard mixed-model calculations to integrate out the random effects (SupplementarySection S2), the log-likelihood of the unknown model parameters given the observed data can be written as

(2) $$L(X,A,B,\sigma^{2}|Y,Z)=-\text{log}\;\det(K)-\text{tr}(K^{-1}C),$$

where

(3) $$K=ZBZ^{T}+ZDX^{T}+XD^{T}Z^{T}+XAX^{T}+\sigma^{2}1$$

and $C=(YY^{T})/m$ is the sample covariance matrix. Maximizing the log-likelihood (2) over positive definite matrices **K** without any further constraints would result in the estimate $\hat{K}=C$ (note that **C** is invertible because we assume that the number of genes *m* is greater than the number of samples *n*; Anderson and Olkin 1985).

If **K** is constrained to be of the form $K=XAX^{T}+\sigma^{2}1$ for a given number of latent factors *p* < *n*, then the model is known as probabilistic PCA and the likelihood is maximized by identifying the latent factors with the eigenvectors of **C** corresponding to the *p* largest eigenvalues (Tipping and Bishop 1999; Lawrence 2005). In matrix form, the probabilistic PCA solution can be written as

(4) $$\hat{K}=P_{1}CP_{1}+{\hat{\sigma }}^{2}P_{2},$$

where $P_{1}$ and $P_{2}$ are mutually orthogonal projection matrices on the space spanned by the first *p* and last *n−p* eigenvectors of **C**, respectively, and the maximum-likelihood estimate ${\hat{\sigma }}^{2}$ is the average variance explained by the *n−p* excluded dimensions (SupplementarySection S5).

If **K** is constrained to be of the form $K=ZBZ^{T}+\sigma^{2}1$, the model is a standard random effects model with the same design matrix **Z** for the random effects *v_i_* for each gene *i*. In general, there exists no analytic solution for the maximum-likelihood estimates of the (co)variance parameter matrix **B** in a random effects model (Gumedze and Dunne 2011). However, in the present context, it is assumed that the data for each gene are an independent sample of the *same* random effects model. Again using the fact that $C=(YY^{T})/m$ is invertible due to the number of genes being greater than the number of samples, the maximum-likelihood solution for **B**, and hence **K**, can be found analytically in terms of **C** and the SVD of **Z**. It turns out to be of the same form (4), except that $P_{1}$ now projects onto the subspace spanned by the known covariates (the columns of **Z**; SupplementarySection S4).

In the most general case where **K** takes the form (3), we show first that every model of the form (1) can be rewritten as a model of the same form where the hidden factors are orthogonal to the known covariates, $X^{T}Z=0$. The reason is that any overlap between the hidden and known covariates can be absorbed in the random effects *v_i_* by a linear transformation and, therefore, simply consists of a reparameterization of the covariance matrices **B** and **D** (SupplementarySection S6). Once this orthogonality is taken into account, the log-likelihood (2) decomposes as a sum $L=L_{1}+L_{2}$, where ${ℒ}_{2}$ is identical to the log-likelihood of probabilistic PCA on the *reduced space* that is the orthogonal complement to the subspace spanned by the known covariates (columns of **Z**). Analogous to the REML method for ordinary linear mixed models, where variance parameters of the random effects are estimated in the subspace orthogonal to the maximum-likelihood estimates of the fixed effects (Patterson and Thompson 1971; Gumedze and Dunne 2011), we estimate the latent variables **X** by maximizing only the likelihood term ${ℒ}_{2}$ corresponding to the subspace where these **X** live (SupplementarySection S6). Once the REML estimates $\hat{X}$ are determined, they become “known” covariates, allowing the covariance parameter matrices to be determined by maximizing the remaining terms ${ℒ}_{1}$ in the likelihood function using the analytic solution for a model with known covariates $\left(Z,\;\hat{X}\right)$ (SupplementarySection S6).

By analogy with the REML method, we call our method the REML method for solving the latent variable model (1), abbreviated “Lvreml”. While the Lvreml solution is not guaranteed to be the absolute maximizer of the total likelihood function, it is guaranteed analytically that for any given number *p* of latent variables, the Lvreml solution attains minimal unexplained variance among all possible choices of *p* latent variables (Supplementary Section S6).

### Lvreml, a flexible software package for learning latent variance components in gene expression data

We implemented the REML method for solving model (1) in a software package Lvreml, available with Matlab and Python interfaces at https://github.com/michoel-lab/lvreml. Lvreml takes as input a gene expression matrix **Y**, a covariate matrix **Z**, and a parameter *ρ*, with 0<ρ<1. This parameter is the desired proportion of variation in **Y** that should be explained by the combined known and latent variance components. Given *ρ*, the number of latent factors *p* is determined automatically (SupplementarySection S7). Lvreml centers the data **Y** such that each sample has mean value zero, to ensure that no fixed effects on the mean need to be included in the model (SupplementarySection S3).

When the number of known covariates (or more precisely the rank of **Z**) exceeds the number of samples, as happens in eQTL studies where a large number of SNPs can act as covariates (Fusi *et al.* 2012), a subset of *n* linearly independent covariates will always explain *all* of the variation in **Y**. In Fusi *et al.* (2012), a heuristic approach was used to select covariates during the likelihood optimization, making it difficult to understand *a priori* which covariates will be included in the model and why. In contrast, Lvreml includes a function to perform initial screening of the covariates, solving for each one the model (1) with a single known covariate to compute the variance ${\hat{\beta }}^{2}$ explained by that covariate alone (SupplementarySection S4). This estimate is then used to include in the final model only those covariates for which ${\hat{\beta }}^{2}\geq \theta \text{tr}(C)$, where θ>0 is the second free parameter of the method, namely the minimum amount of variation a known covariate needs to explain on its own to be included in the model (SupplementarySection S7). In the case of genetic covariates, we further propose to apply this selection criterion not to individual SNPs, but to principal components (PCs) of the genotype data matrix. Since PCA is a linear transformation of the genotype data, it does not alter model (1). Moreover, selecting PCs as covariates ensures that the selected covariates are linearly independent and are consistent with the fact that genotype PCs are known to reveal population structure in expression data (Brown *et al.* 2018).

**Figure 1:**
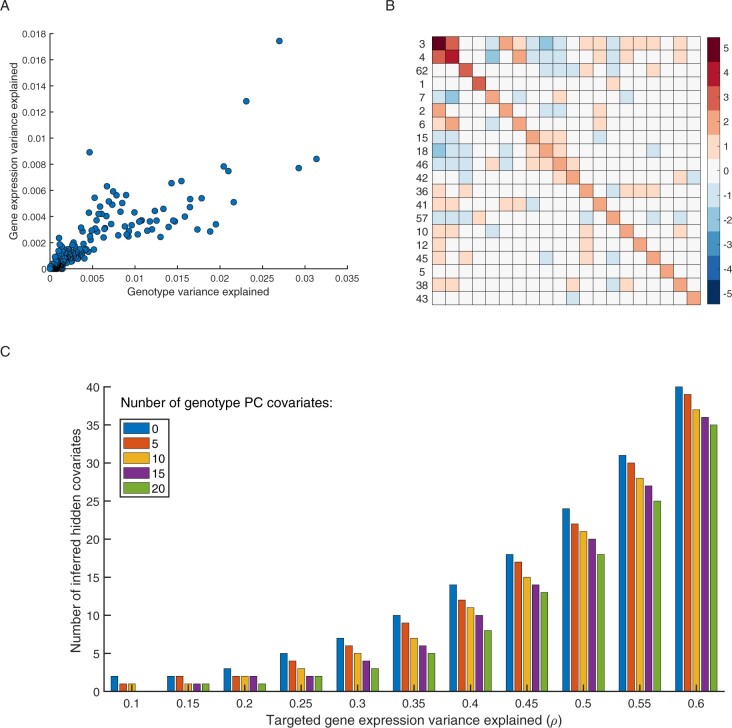
(A) Gene expression variance explained by individual genotype PCs in univariate models *vs* their genotype variance explained. (B) Heatmap of the estimated covariance matrix **B** [cf. (3)] among the effects on gene expression of the top 20 genotype PCs (by gene expression variance explained in univariate models, cf. A, *y*-axis); the row labels indicate the genotype PC index, ranked by genotype variance explained (cf. A, *x*-axis). (C) Number of hidden covariates inferred by Lvreml as a function of the parameter *ρ* (the targeted total amount of variance explained by the known and hidden covariates), with *θ* (the minimum variance explained by a known covariate) set to retain 0, 5, 10, or 20 known covariates (genotype PCs) in the model. For visualization purposes only the range of *ρ* upto ρ=0.6 is shown, for the full range, see Supplementary Figure S1.

To test Lvreml and illustrate the effect of its parameters, we used genotype data for 42,052 genetic markers and RNA sequencing expression data for 5720 genes in 1012 segregants from a cross between two strains of budding yeast (Albert *et al.* 2018), one of the largest (in terms of sample size), openly available eQTL studies in any organism (see *Materials and methods*). We first performed PCA on the genotype data. The dominant genotype PCs individually explained 2–3% of variation in the genotype data, and 1–2% of variation in the expression data, according to the single-covariate model [Supplementary Section S4, Supplementary Equation (S16), and Figure 1A]. Although genotype PCs are orthogonal by definition, their effects on gene expression are not independent, as shown by the non-zero off-diagonal entries in the maximum-likelihood estimate of the covariance matrix **B** [cf. (3); Figure 1B]. To illustrate how the number of inferred hidden covariates varies as a function of the input parameter *ρ*, we determined values of the parameter *θ* to include between 0 and 20 genotype PCs as covariates in the model. As expected, for a fixed number of known covariates, the number of hidden covariates increases with *ρ*, as more covariates are needed to explain more of the variation in **Y**, and decreases with the number of known covariates, as fewer hidden covariates are needed when the known covariates already explain more of the variation in **Y** (Figure 1C).

When setting the parameter *θ*, or equivalently, deciding the number of known covariates to include in the model, care must be taken due to a mathematical property of the model: the maximizing solution exists only if the minimum amount of variation in **Y** explained by a known covariate (or more precisely, by a principal axis in the space spanned by the known covariates) is greater than the maximum-likelihood estimate of the residual variance ${\hat{\sigma }}^{2}$ (see Theorems 1 and 4 in SupplementarySections S4 and S6). If noninformative variables are included among the known covariates, or known covariates are strongly correlated, then the minimum variation explained by them becomes small, and potentially smaller than the residual variance, whose initial “target” value is 1−ρ. Because Lvreml considers the known covariates as fixed, it lowers the value of ${\hat{\sigma }}^{2}$ by including more hidden covariates in the model, until the existence condition is satisfied. In such cases, the total variance explained by the known and hidden covariates will be greater than the target value of the input parameter *ρ*. Visually, the presence of noninformative dimensions in the linear subspace spanned by the known covariates (due to noninformative or redundant variables) is shown by a saturation of the number of inferred hidden covariates with decreasing *ρ* (SupplementaryFigureS1B), providing a clear cue that the relevance or possible redundancy of (some of) the known covariates for explaining variation in the expression data needs to be reconsidered.

### Lvreml attains likelihood values higher than or equal to Panama

**Figure 2:**
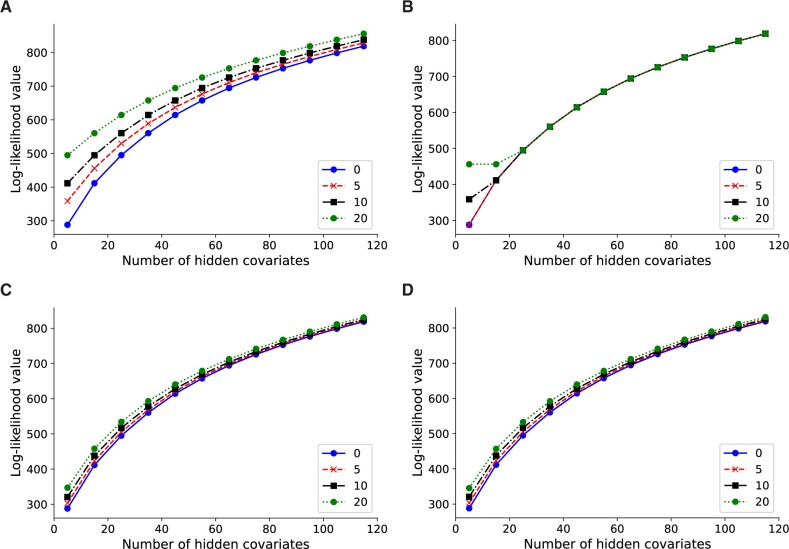
Log-likelihood values for Lvreml (A, C) and Panama (B, D) using 0, 5, 10, and 20 PCs of the expression data (A, B) or genotype data (C, D) as known covariates. The results shown are for 600 randomly subsampled segregants; corresponding results for 200, 400, and in the case of Lvreml 1012 segregants are shown in Supplementary Figure S2.

To compare the analytic solution of Lvreml against the original model with gradient-based optimization algorithm, as implemented in the Panama software (Fusi *et al.* 2012), we performed a controlled comparison where 0, 5, 10, and 20 dominant PCs of the expression data **Y** were used as artificial known covariates. Because of the mathematical properties of the model and the Lvreml solution, if the first *d* expression PCs are included as known covariates, Lvreml will return the next *p* expression PCs as hidden factors. Hence, the log-likelihood of the Lvreml solution with *d* expression PCs as known covariates and *p* hidden factors will coincide with the log-likelihood of the solution with zero known covariates and *d* + *p* hidden factors (that is, probabilistic PCA with *d* + *p* hidden factors). Figure 2A shows that this is the case indeed: the log-likelihood curves for 0, 5, 10, and 20 PCs as known covariates are shifted horizontally by a difference of exactly 5 (from 0, to 5, to 10) or 10 (from 10 to 20) hidden factors.

In contrast, Panama did not find the optimal shifted probabilistic PCA solution, and its likelihood values largely coincided with the solution with zero known covariates, irrespective of the number of known covariates provided (Figure 2B). In other words, Panama did not use the knowledge of the known covariates to explore the orthogonal space of axes of variation not yet explained by the known covariates, instead arriving at a solution where *p* hidden factors appear to explain no more of the variation than *p−d* PCs orthogonal to the *d* known PCs. To verify this, we compared the Panama hidden factors to PCs given as known covariates, and found that in all cases where the curves in Figure 2B align, the first *d* hidden factors coincided indeed with the *d* known covariates (data not shown).

When genotype PCs were used as known confounders (using the procedure explained above), the shift in log-likelihood values was less pronounced, consistent with the notion that the genotype PCs explain less of the expression variation than the expression PCs. In this case, the likelihood values of Lvreml and Panama coincided (Figure 2, C and D), indicating that both methods found the same optimal covariance matrix.

The explanation for the difference between Figure 2, A and C is as follows. In Figure 2A, Lvreml uses *p* hidden covariates to explain the same amount of variation as *d* + *p* expression PCs. The dominant expression PCs are partially explained by population structure (genotype data). Hence, when *d* genotype PCs are given as known covariates, Lvreml infers *p* orthogonal latent variables that explain the “missing” portions of the expression PCs not explained by genotype data. This results in a model that explains more expression variation than the *p* dominant expression PCs, but less than *p* + *d* expression PCs, hence the reduced shift in Figure 2C.

It is unclear why Panama did not find the correct solution when expression PCs were used as known covariates (Figure 2B), but this behavior was consistent across multiple subsampled datasets of varying sizes (SupplementaryFigureS2) as well as in other datasets (data not shown).

### Panama and Peer infer hidden factors that are partially redundant with the known covariates

Although Panama inferred models with the same covariance matrix estimate $\hat{K}$ and hence the same likelihood values as Lvreml when genotype PCs where given as known covariates, the inferred hidden covariates differed between the methods.

**Figure 3:**
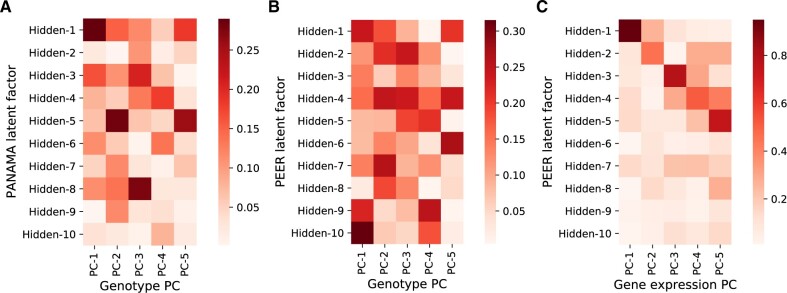
Cosine similarity between known covariates (five genotype PCs) given to the model and hidden factors inferred by Panama (A) and Peer (B), and cosine similarity between gene expression PCs and hidden factors inferred by Peer (C) when no known covariates are given to the model. Results are for randomly subsampled data of 200 segregants.

As explained, hidden covariates inferred by Lvreml are automatically orthogonal to the known covariates and represent linearly independent axes of variation. In contrast, the latent variables inferred by Panama overlapped with the known genotype covariates supplied to the model, with cosine similarities of up to 30% (Figure 3A). In Panama, covariances among the effects of the known confounders are assumed to be zero. When the optimal model (*i.e.*, maximum-likelihood $\hat{K}$) in fact has effects with non-zero covariance (as in Figure 1B), the optimization algorithm in Panama will automatically select hidden confounders that overlap with the known confounders to account for these non-zero covariances (SupplementarySection S6), thus resulting in the observed overlap. Hence, the common interpretation of Panama factors as new determinants of gene expression distinct from known genetic factors is problematic.

To test whether the overlap between inferred and already known covariates also occurs in other methods or is specific to Panama, we ran the Peer software (Stegle *et al.* 2012) on a reduced dataset of 200 randomly selected samples from the yeast data (Peer runtimes made it infeasible to run on larger sample sizes). Peer is a popular software that uses a more elaborate hierarchical model to infer latent variance components (Stegle *et al.* 2010). Peer hidden factors again showed cosine similarities of up to 30% (Figure 3B), suggesting that its hidden factors also cannot be interpreted as completely new determinants of gene expression. We also tested the hidden factors returned by Peer when no known covariates are added to the model. In this case, model (1) reduces to probabilistic PCA and both Lvreml and Panama correctly identify the dominant expression PCs as hidden factors (Figure 2, A and B). Despite its more complex model, which does not permit an analytic solution even in the absence of known covariates, Peer hidden factors in fact do overlap strongly with the same dominant expression PCs (cosine similarities between 60% and 80%), indicating that the added value of the more complicated model structure may be limited, at least in this case.

### Lvreml is orders of magnitude faster than Panama

**Figure 4:**
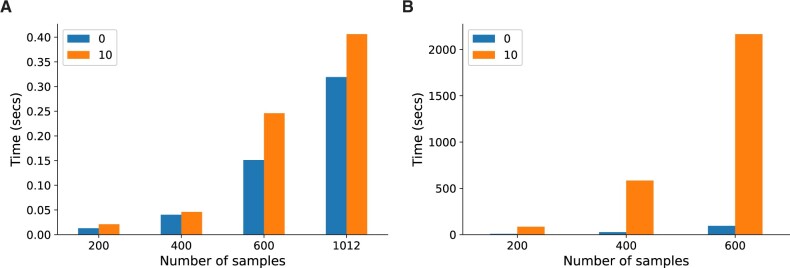
Runtime comparison between Lvreml (A) and Panama (B), with parameters set to infer 85 hidden covariates with either 0 known covariates or including 10 genotype PCs as known covariates, at multiple sample sizes. Running Panama on the full dataset of 1012 segregants was infeasible. For runtime comparisons at other parameter settings, see Supplementary Figure S3.

An analytic solution does not only provide additional insight into the mathematical properties of a model but can also provide significant gains in computational efficiency. The Lvreml solution can be computed using standard matrix operations from linear algebra, for which highly optimized implementations exist in all programming languages. Comparison of the runtime of the Python implementations of Lvreml and Panama on the yeast data at multiple sample sizes showed around 10 thousand-fold speed-up factors, from several minutes for a single Panama run to a few tens of milliseconds for Lvreml (Figure 4). Interestingly, the computational cost of Lvreml did not increase much when known covariates were included in the model, compared to the model without known covariates that is solved by PCA (Figure 4A). In contrast, runtime of Panama blows up massively as soon as covariates are included (Figure 4B). Nevertheless, even in the case of no covariates, Panama is around 600 times slower than the direct, eigenvector decomposition-based solution implemented in Lvreml. Finally, the runtime of Lvreml does not depend on the number of known or inferred latent factors, whereas increasing either parameter in Panama leads to an increase in runtime (SupplementaryFigureS3).

## Discussion

We presented a random effects model to estimate simultaneously the contribution of known and latent variance components in gene expression data, which is closely related to models that have been used previously in this context (Lawrence 2005; Stegle *et al.* 2010, 2012; Fusi *et al.* 2012; Buettner *et al.* 2015). By including additional parameters in our model to account for nonzero covariances among the effects of known covariates and latent factors, we were able to show that latent factors can always be taken orthogonal to, and therefore linearly independent of, the known covariates supplied to the model. This is important, because inferred latent factors are not only used to correct for correlation structure in the data but also as new, data-derived “endophenotypes”, that is, determinants of gene expression whose own genetic associations are biologically informative (Parts *et al.* 2011; Stegle *et al.* 2012). As shown in this paper, the existing models and their numerical optimization result in hidden factors that in fact overlap significantly with the known covariates, and hence their value in uncovering “new” determinants of gene expression must be questioned.

To solve our model, we did not rely on numerical, gradient-based optimizers, but rather on an analytic REML solution. This solution relies on a decomposition of the log-likelihood function that allows us to identify hidden factors as PCs of the expression data matrix reduced to the orthogonal complement of the subspace spanned by the known covariates. This solution is guaranteed to minimize the amount of unexplained variation in the expression data for a given number of latent factors and is analogous to the widely used REML solution for conventional linear mixed models, where variance parameters of random effects are estimated in the subspace orthogonal to the maximum-likelihood estimates of the fixed effects.

Having an analytic solution is not only important for understanding the mathematical properties of a statistical model, but can also lead to significant reduction of the computational cost for estimating parameter values. Here, we obtained a 10,000-fold speed-up compared to an existing software that uses gradient-based optimization. On a yeast dataset with 1012 samples, our method could solve the covariance structure and infer latent factors in less than half a second, whereas it was not feasible to run an existing implementation of gradient-based optimization on more than 600 samples.

The experiments on the yeast data showed that in real-world scenarios, Lvreml and the gradient-based optimizer implemented in the Panama software resulted in the same estimates for the sample covariance matrix. Although the latent variables inferred by both methods are different (orthogonal *vs* partially overlapping with the population structure covariates), we anticipate that downstream linear association analyses will nevertheless give similar results as well. For instance, established protocols (Stegle *et al.* 2012) recommend to use known and latent factors as covariates to increase the power to detect expression QTLs. Since orthogonal and overlapping latent factors can be transformed into each other through a linear combination with the known confounders, linear association models that use both known and latent factors as covariates will also be equivalent (SupplementarySection S8).

While we have demonstrated that the use of latent variance components that are orthogonal to known confounders leads to significant analytical and numerical advantages, we acknowledge that it follows from a mathematical symmetry of the underlying statistical model that allows us to transform a model with overlapping latent factors to an equivalent model with orthogonal factors. Whether the true but unknown underlying variance components are orthogonal or not, nor their true overlap value with the known confounders, can be established by the models studied in this paper precisely due to this mathematical symmetry. Such limitations are inherent to all latent variable methods.

To conclude, we have derived an analytic REML solution for a widely used class of random effects models for learning latent variance components in gene expression data with known and unknown confounders. Our solution can be computed in a highly efficient manner, identifies hidden factors that are orthogonal to the already known variance components, and results in the estimation of a sample covariance matrix that can be used for the downstream estimation of variance parameters for individual genes. The REML method facilitates the application of random effects modeling strategies for learning latent variance components to much larger gene expression datasets than currently possible.

## Data availability

The Lvreml software and all data processing and analysis scripts underlying this article are available at https://github.com/michoel-lab/lvreml.

The modified code for running the Panama analyses is available as a fork of the Limix package at https://github.com/michoel-lab/limix-legacy.

No new data were generated in support of this research.

Expression levels in units of log2(TPM) for all yeast genes and segregants were obtained from https://doi.org/10.7554/eLife.35471.021.

Information on experimental batch and growth covariates for all yeast segregants was obtained from https://doi.org/10.7554/eLife.35471.022.

Genotypes at 42,052 markers for all yeast segregants were obtained from https://doi.org/10.7554/eLife.35471.023.

Supplementary material is available at *G3* online.

## Funding

This research was supported in part by a grant from the Research Council of Norway (grant number 312045) to T.M.

## Conflicts of interest

The authors declare that there is no conflict of interest.

## Supplementary Material

jkab410_Supplementary_Data
