# Supplementary material for: Restricted maximum-likelihood method for learning latent variance components in gene expression data with known and unknown confounders
Source: G3 (Bethesda). 2021 Dec 1;12(2):jkab410. doi: 10.1093/g3journal/jkab410 (PMC9210293; doi:10.1093/g3journal/jkab410)
Supplement: jkab410_Supplementary_Data [file jkab410_supplementary_data.pdf]

Restricted maximum-likelihood method for learning latent variance components in gene expression data with known and unknown confounders

— Supplementary Information —

Muhammad Ammar Malik and Tom Michoel*

Computational Biology Unit, Department of Informatics, University of Bergen, PO Box 7803, 5020 Bergen, Norway

* Corresponding author, email: tom.michoel@uib.no

Supplementary Figures

A

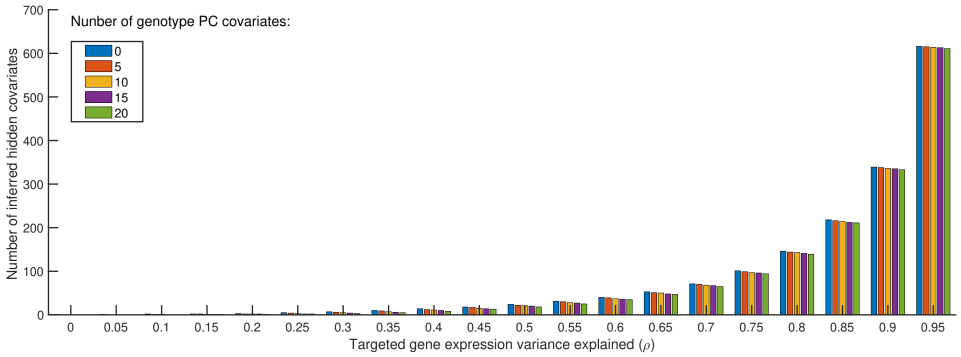

B

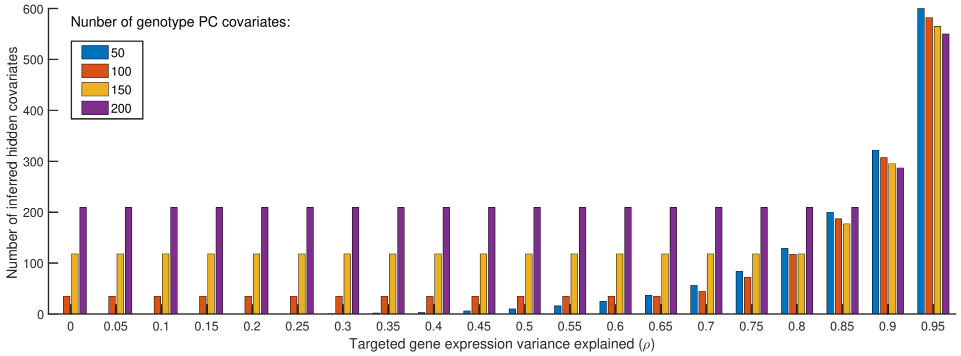

Figure S1: **A.** Number of hidden covariates inferred by LVREML as a function of the parameter ρ (the targeted total amount of variance explained by the known and hidden covariates), with θ (the minimum variance explained by a known covariate) set to retain 0, 5, 10, or 20 known covariates (genotype PCs) in the model. **B.** Same as panel **A**, with θ set to retain 50, 100, 150, or 200 genotype PCs in the model. The saturation of the number of hidden covariates with decreasing ρ for models with 100, 150, and 200 known covariates is a visual indicator that some of the dimensions in the linear subspace spanned by the known covariates do not explain sufficient variation in the expression data, and the relevance or possible redundancy of (some of) the known covariates for explaining variation in the expression data needs to be reconsidered.

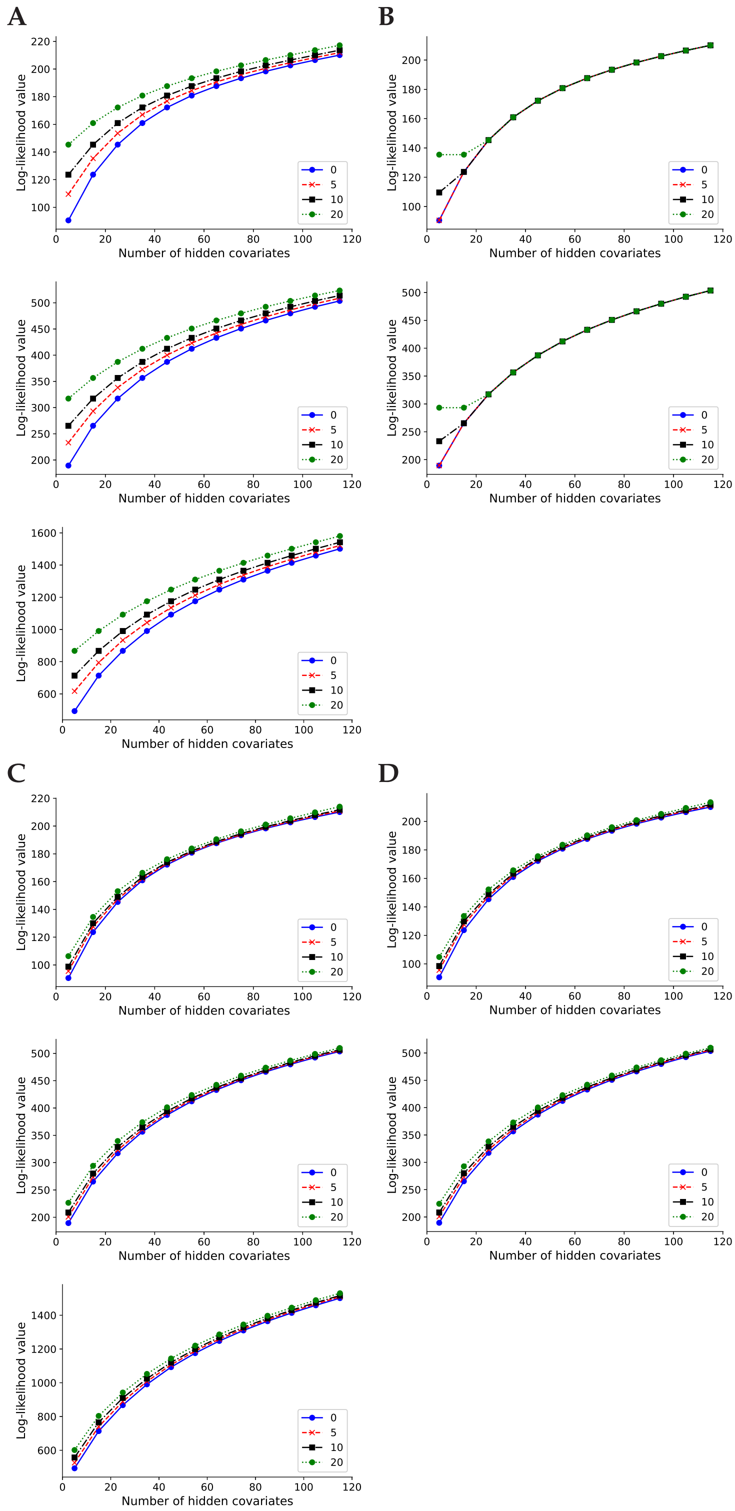

Figure S2: Log-likelihood values for LVREML (**A**,**C**) and PANAMA (**B**,**D**) using 0, 5, 10, and 20 PCs of the expression data (**A**,**B**) or genotype data (**C**,**D**) as known covariates, at sample sizes of 200, 400, and in the case of LVREML 1,012 segregants (top to bottom).

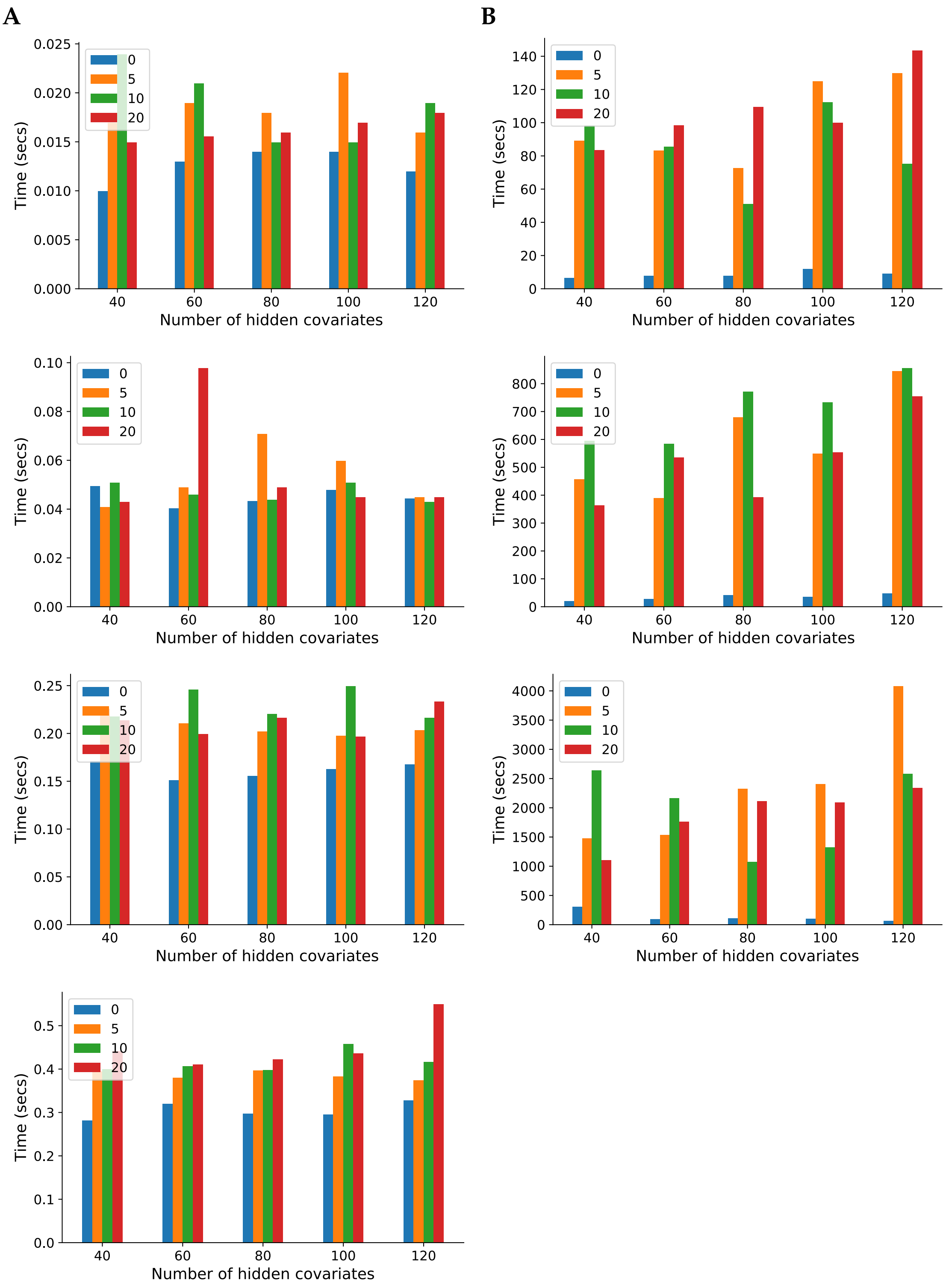

Figure S3: Runtime comparison on between LVREML (**A**) and PANAMA (**B**), with parameters set to infer 85 hidden covariates with 0, 5, 10, or 20 genotype PCs included as known covariates, at sample sizes of 200, 400, and in the case of LVREML 1,012 segregants (top to bottom).

Supplementary Methods

S1 Preliminary results

In the sections below, we will repeatedly use the following results. The first result concerns linear transformations of normally distributed variables and can be found in most textbooks on statistics or probability theory:

Lemma 1. *Let $x \in \mathbb{R}^n$ be a random, normally distributed vector,*

$$p(x) = \mathcal{N}(\mu, \Psi),$$

with $\mu \in \mathbb{R}^n$, and $\Psi \in \mathbb{R}^{n\times n}$ a positive definite covariance matrix. For any linear transformation $y = \mathbf{M}x$ with $\mathbf{M} \in \mathbb{R}^{m\times n}$, we have

$$p(y) = \mathcal{N}(\mathbf{M}\mu, \mathbf{M}\Psi\mathbf{M}^T).$$

□

If the linear transformation $y = \mathbf{M}x$ in this Lemma is overdetermined, that is, if $m > n$, then the transformed covariance matrix $\Psi' = \mathbf{M}\Psi\mathbf{M}^T$ will have a lower rank n than its dimension m, that is, $\Psi' \in \mathbb{R}^{m\times m}$ is a positive *semi*-definite matrix (i.e., has one or more zero eigenvalues). Thus we can extend the definition of normal distributions to include *degenerate* distributions with positive *semi*-definite covariance matrix, by interpreting them as the distributions of overdetermined linear combinations of normally distributed vectors. A degenerate one-dimensional normal distribution is simply defined as a δ-distribution, that is, for $x \in \mathbb{R}$

$$p(x) = \mathcal{N}(\mu, 0) = \delta(x - \mu),$$

which can be derived as a limit $\sigma^2 \to 0$ of normal distribution density functions $\mathcal{N}(\mu, \sigma^2)$.

The second result is one that is attributed to von Neumann [1]:

Lemma 2. *Let $\mathbf{P}, \mathbf{Q} \in \mathbb{R}^{n\times n}$ be two positive definite matrices. Then*

$$\mathrm{tr}(\mathbf{P}^{-1}\mathbf{Q}) \geq \sum_{i=1}^{n} \pi_i^{-1}\chi_i, \tag{S1}$$

where $\pi_1 \geq \cdots \geq \pi_n$ and $\chi_1 \geq \cdots \geq \chi_n$ are the ordered eigenvalues of $\mathbf{P}$ and $\mathbf{Q}$, respectively, and equality in eq. (S1) *is achieved if and only if the eigenvector of $\mathbf{P}$ corresponding to π_i is equal to the eigenvector of $\mathbf{Q}$ corresponding to χ_{n-i+1}, $i = 1, \dots, n$.* □

S2 The model

We will use the following notation:

- $\mathbf{Y} \in \mathbb{R}^{n \times m}$ is a matrix of gene expression data for m genes in n samples. The ith column of $\mathbf{Y}$ is denoted $y_i \in \mathbb{R}^n$ and corresponds to the vector of expression values for gene i. We assume that the data in each sample are centred, $\sum_{i=1}^m y_i = 0 \in \mathbb{R}^n$.
- $\mathbf{Z} \in \mathbb{R}^{n \times d}$ is a matrix of values for d known confounders in the same n samples. The kth column of $\mathbf{Z}$ is denoted $z_k \in \mathbb{R}^n$ and corresponds to the data for confounding factor k.
- $\mathbf{X} \in \mathbb{R}^{n \times p}$ is a matrix of values for p latent variables to be determined in the same n samples. The jth column of $\mathbf{X}$ is denoted $x_j \in \mathbb{R}^n$.

To identify the hidden correlation structure of the expression data, we assume a linear relationship between expression levels and the known and latent variables, with random noise added:

$$y_i = \mathbf{Z} v_i + \mathbf{X} w_i + \epsilon_i, \tag{S2}$$

where $v_i \in \mathbb{R}^d$ and $w_i \in \mathbb{R}^p$ are jointly normally distributed random vectors,

$$p\left(\begin{bmatrix} v_i \\ w_i \end{bmatrix}\right) = \mathcal{N}\left(0, \begin{bmatrix} \mathbf{B} & \mathbf{D} \\ \mathbf{D}^T & \mathbf{A} \end{bmatrix}\right) \tag{S3}$$

with $\mathbf{B} \in \mathbb{R}^{d \times d}$, $\mathbf{D} \in \mathbb{R}^{d \times p}$ and $\mathbf{A} = \mathrm{diag}(\alpha_1^2, \dots, \alpha_p^2)$, such that

$$\Psi = \begin{bmatrix} \mathbf{B} & \mathbf{D} \\ \mathbf{D}^T & \mathbf{A} \end{bmatrix}$$

is a positive semi-definite matrix; the errors $\epsilon_i \in \mathbb{R}^n$ are assumed to be independent and normally distributed,

$$p(\epsilon_i) = \mathcal{N}(0, \sigma^2 \mathbb{1}).$$

Note that our aim is to identify variance components shared across genes, and hence σ^2 is assumed to be the same for all i. By assumption, the errors are also independent of the effect sizes, and hence we can write

$$p\left(\begin{bmatrix} v_i \\ w_i \\ \epsilon_i \end{bmatrix}\right) = \mathcal{N}\left(0, \begin{bmatrix} \mathbf{B} & \mathbf{D} & 0 \\ \mathbf{D}^T & \mathbf{A} & 0 \\ 0 & 0 & \sigma^2 \mathbb{1} \end{bmatrix}\right). \tag{S4}$$

By Lemma 1, y_i is normally distributed with distribution

$$p(y_i) = \mathcal{N}(0, \mathbf{K}) = \frac{1}{(2\pi)^{\frac{n}{2}} \sqrt{\det(\mathbf{K})}} \exp\left(-\frac{1}{2} \langle y_i, \mathbf{K}^{-1} y_i \rangle\right), \tag{S5}$$

where

$$\mathbf{K} = \begin{bmatrix} \mathbf{Z} & \mathbf{X} & \mathbb{1} \end{bmatrix} \begin{bmatrix} \mathbf{B} & \mathbf{D} & 0 \\ \mathbf{D}^T & \mathbf{A} & 0 \\ 0 & 0 & \sigma^2 \mathbb{1} \end{bmatrix} \begin{bmatrix} \mathbf{Z}^T \\ \mathbf{X}^T \\ \mathbb{1} \end{bmatrix} = \mathbf{Z}\mathbf{B}\mathbf{Z}^T + \mathbf{Z}\mathbf{D}\mathbf{X}^T + \mathbf{X}\mathbf{D}^T\mathbf{Z} + \mathbf{X}\mathbf{A}\mathbf{X}^T + \sigma^2 \mathbb{1},$$

and we used the notation $\langle u, v \rangle = u^T v$ to denote the inner product between two vectors in $\mathbb{R}^n$.

Defining matrices $\mathbf{V} \in \mathbb{R}^{d \times m}$ and $\mathbf{W} \in \mathbb{R}^{p \times m}$, whose columns are the random effect vectors v_i and w_i, respectively, eq. (S2) can be written in matrix notation as

$$\mathbf{Y} = \mathbf{ZV} + \mathbf{XW} + \epsilon$$

Under the assumption that the columns y_i of $\mathbf{Y}$ are independent samples of the distribution (S5), the likelihood of observing $\mathbf{Y}$ given covariate data $\mathbf{Z}$, (unknown) latent variable data $\mathbf{X}$ and values for the hyper-parameters $\Theta = \{\sigma^2, \mathbf{A}, \mathbf{B}, \mathbf{D}\}$, is given by

$$p(\mathbf{Y} \mid \mathbf{Z}, \mathbf{X}, \Theta) = \prod_{i=1}^{m} p(y_i \mid 0, \mathbf{K}).$$

Note that in standard mixed-model calculations, the distribution (S5) is often arrived at by integrating out the random effects. This is equivalent to application of Lemma 1.

To conclude, the log-likelihood is, upto an additive constant, and divided by half the number of genes:

$$\mathcal{L} = -\frac{2}{m}\Big[\frac{m}{2}\log\det(\mathbf{K}) + \frac{1}{2}\sum_{i=1}^{m}\langle y_i, \mathbf{K}^{-1} y_i\rangle\Big] = -\log\det(\mathbf{K}) - \operatorname{tr}\big(\mathbf{K}^{-1}\mathbf{C}\big),$$

where

$$\mathbf{C} = \frac{\mathbf{Y}\mathbf{Y}^T}{m}$$

is the empirical covariance matrix.

S3 Systematic effects on the mean

Eq. (S2) only considers random effects, which leads to a model for studying systematic effects on the covariance between samples. We could also include fixed effects to model systematic effects on mean expression level. However, by centering the data, $\sum_{i=1}^{m} y_i = 0$, the maximum-likelihood estimate of such fixed effects is always zero. To see this, let $\mathbf{T} \in \mathbb{R}^{n \times c}$ be a matrix of c covariates with fixed effects $\beta \in \mathbb{R}^c$ shared across genes (we are only interested in discovering systematic biases in the data). Then the minus log-likelihood (2) becomes

$$\mathcal{L} = \log\det(\mathbf{K}) + \frac{1}{m}\sum_{i=1}^{m}\langle y_i - \mathbf{T}\beta, \mathbf{K}^{-1}(y_i - \mathbf{T}\beta)\rangle$$

Optimizing with respect to β leads to the equation

$$\hat{\beta} = (\mathbf{T}^T\mathbf{K}^{-1}\mathbf{T})^{-1}\mathbf{T}^T\bar{y}$$

where

$$\bar{y} = \frac{1}{m}\sum_{i=1}^{m} y_i = 0.$$

S4 Solution of the model without latent variables

We start by considering the problem of finding the maximum-likelihood solution in the absence of any latent variables, i.e. minimizing eq. (2) with

$$\mathbf{K} = \mathbf{Z}\mathbf{B}\mathbf{Z}^T + \sigma^2 \mathbb{1} \tag{S6}$$

with respect to $\mathbf{B}$ and σ^2.

Note first of all that we may assume the set of confounding factors $\{z_1, \dots, z_d\}$ to be linearly independent, because if not, the expression in eq. (S2) can be rearranged in terms of a linearly independent subset of factors whose coefficients are still normally distributed due to elementary properties of the multivariate normal distribution, see for instance the proof of Lemma 5 below. Linear independence of $\{z_1, \dots, z_d\}$ implies that we must have $d \leq n$ and $\text{rank}(\mathbf{Z}) = d$.

The singular value decomposition allows to decompose $\mathbf{Z}$ as $\mathbf{Z} = \mathbf{U}\mathbf{\Gamma}\mathbf{V}^T$, where $\mathbf{U} \in \mathbb{R}^{n\times n}$, $\mathbf{U}^T\mathbf{U} = \mathbf{U}\mathbf{U}^T = \mathbb{1}$, $\mathbf{\Gamma} \in \mathbb{R}^{n\times d}$ diagonal with $\gamma_k^2 \equiv \Gamma_{kk} > 0$ for $k \in \{1, \dots, d\}$ [this uses $\text{rank}(\mathbf{Z}) = d$], and $\mathbf{V} \in \mathbb{R}^{d\times d}$, $\mathbf{V}^T\mathbf{V} = \mathbf{V}\mathbf{V}^T = \mathbb{1}$. There is also a 'thin' SVD, $\mathbf{Z} = \mathbf{U}_1\mathbf{\Gamma}_1\mathbf{V}^T$, where $\mathbf{U}_1 \in \mathbb{R}^{n\times d}$, $\mathbf{U}_1^T\mathbf{U}_1 = \mathbb{1}$, $\mathbf{\Gamma}_1 \in \mathbb{R}^{d\times d}$ diagonal with diagonal elements γ_k^2. In block matrix notation, $\mathbf{U} = (\mathbf{U}_1, \mathbf{U}_2)$ and

$$\mathbf{Z} = \begin{pmatrix} \mathbf{U}_1 & \mathbf{U}_2 \end{pmatrix} \begin{pmatrix} \mathbf{\Gamma}_1 \\ 0 \end{pmatrix} \mathbf{V} \tag{S7}$$

Note that unitarity of $\mathbf{U}$ implies $\mathbf{U}_1^T\mathbf{U}_2 = 0$.

Denote by $\mathcal{H}_Z$ the space spanned by the columns (i.e. covariate vectors) of $\mathbf{Z}$. The projection matrix $\mathbf{P}_Z$ onto $\mathcal{H}_Z$ is given by

$$\mathbf{P}_Z = \mathbf{Z}(\mathbf{Z}^T\mathbf{Z})^{-1}\mathbf{Z}^T = \mathbf{U}_1\mathbf{\Gamma}_1\mathbf{V}^T(\mathbf{V}\mathbf{\Gamma}_1^{-2}\mathbf{V}^T)\mathbf{V}\mathbf{\Gamma}_1\mathbf{U}_1^T = \mathbf{U}_1\mathbf{U}_1^T.$$

Using the basis of column vectors of $\mathbf{U}$, we can write any matrix $\mathbf{M} \in \mathbb{R}^{n\times n}$ as a partitioned matrix

$$\mathbf{U}^T\mathbf{M}\mathbf{U} = \begin{pmatrix} \mathbf{M}_{11} & \mathbf{M}_{12} \\ \mathbf{M}_{21} & \mathbf{M}_{22} \end{pmatrix} \tag{S8}$$

where

$$\mathbf{M}_{ij} = \mathbf{U}_i^T\mathbf{M}\mathbf{U}_j. \tag{S9}$$

The following results for partitioned matrices are derived easily or can be found in [2]:

$$\text{tr}(\mathbf{M}) = \text{tr}(\mathbf{M}_{11}) + \text{tr}(\mathbf{M}_{22}) \tag{S10}$$

$$\det(\mathbf{M}) = \det\big(\mathbf{M}_{11} - \mathbf{M}_{12}\mathbf{M}_{22}^{-1}\mathbf{M}_{21}\big)\ \det(\mathbf{M}_{22}) \tag{S11}$$

Using this notation, the following result solves the model without latent variables:

Theorem 1. *Let $\mathbf{C} \in \mathbb{R}^{n\times n}$ be a positive definite matrix such that*

$$\lambda_{\min}(\mathbf{C}_{11}) > \frac{\operatorname{tr}(\mathbf{C}_{22})}{n-d}, \tag{S12}$$

where $\lambda_{\min}(\cdot)$ denotes the smallest eigenvalue of a matrix. Then the maximum-likelihood solution

$$\hat{\mathbf{K}} = \underset{\{\mathbf{K}:\ \mathbf{K}=\mathbf{ZBZ}^T+\sigma^2\mathbb{1}\}}{\operatorname{argmin}} \log\det\mathbf{K} + \operatorname{tr}\big(\mathbf{K}^{-1}\mathbf{C}\big), \tag{S13}$$

subject to $\mathbf{B}$ being positive semi-definite and $\sigma^2 \geq 0$, is given by

$$\hat{\mathbf{B}} = \mathbf{V}\boldsymbol{\Gamma}_1^{-1}(\mathbf{C}_{11} - \hat{\sigma}^2\mathbb{1})\boldsymbol{\Gamma}_1^{-1}\mathbf{V}^T \tag{S14}$$

$$\hat{\sigma}^2 = \frac{\operatorname{tr}(\mathbf{C}_{22})}{n-d} \tag{S15}$$

Proof. Using eq. (S7), we can write

$$\begin{aligned}\mathbf{K} = \mathbf{ZBZ}^T + \sigma^2\mathbb{1} &= \mathbf{U}_1\boldsymbol{\Gamma}_1\mathbf{V}^T\mathbf{B}\mathbf{V}\boldsymbol{\Gamma}_1\mathbf{U}_1^T + \sigma^2(\mathbf{U}_1\mathbf{U}_1^T + \mathbf{U}_2\mathbf{U}_2^T)\\ &= \mathbf{U}_1\boldsymbol{\Gamma}_1\mathbf{V}^T\big(\mathbf{B} + \sigma^2\mathbf{V}\boldsymbol{\Gamma}_1^{-2}\mathbf{V}^T\big)\mathbf{V}\boldsymbol{\Gamma}_1\mathbf{U}_1^T + \sigma^2\mathbf{U}_2\mathbf{U}_2^T.\end{aligned}$$

Hence, in the block matrix notation (S8), we have

$$\begin{aligned}\mathbf{K}_{11} &= \boldsymbol{\Gamma}_1\mathbf{V}^T\big(\mathbf{B} + \sigma^2\mathbf{V}\boldsymbol{\Gamma}_1^{-2}\mathbf{V}^T\big)\mathbf{V}\boldsymbol{\Gamma}_1\\ \mathbf{K}_{22} &= \sigma^2\mathbb{1}\\ \mathbf{K}_{12} &= \mathbf{K}_{21} = 0.\end{aligned}$$

It follows that

$$\mathbf{K}^{-1} = \begin{pmatrix}\mathbf{K}_{11}^{-1} & 0\\ 0 & \mathbf{K}_{22}^{-1}\end{pmatrix}$$

and, using eqs. (S10) and (S11),

$$\begin{aligned}\log\det(\mathbf{K}) &= \log\det(\mathbf{K}_{11}) + \log\det(\mathbf{K}_{22}) = \log\det(\mathbf{K}_{11}) + (n-d)\log(\sigma^2)\\ \operatorname{tr}(\mathbf{K}^{-1}\mathbf{C}) &= \operatorname{tr}(\mathbf{K}_{11}^{-1}\mathbf{C}_{11}) + \operatorname{tr}(\mathbf{K}_{22}^{-1}\mathbf{C}_{22}) = \operatorname{tr}(\mathbf{K}_{11}^{-1}\mathbf{C}_{11}) + \frac{\operatorname{tr}(\mathbf{C}_{22})}{\sigma^2}.\end{aligned}$$

Let $\mathbf{C}_{11}$ have eigenvalues $\lambda_1 \geq \cdots \geq \lambda_d$ with corresponding eigenvectors $u_1, \ldots, u_d \in \mathbb{R}^d$. Applying Lemma 2 to the term $\operatorname{tr}(\mathbf{K}_{11}^{-1}\mathbf{C}_{11})$, it follows that for the minimizer $\hat{\mathbf{K}}$, $\hat{\mathbf{K}}_{11}$ must have eigenvalues $\kappa_1 \geq \cdots \geq \kappa_d$ with the same eigevectors $u_1, \ldots, u_d$ as $\mathbf{C}_{11}$. Expressing the minus log-likelihood in terms of these eigenvalues results in

$$\mathcal{L}(\hat{\mathbf{K}}) = \sum_{i=1}^{d}\log(\kappa_i) + \sum_{i=1}^{d}\kappa_i^{-1}\lambda_i + (n-d)\log(\sigma^2) + \frac{\operatorname{tr}(\mathbf{C}_{22})}{\sigma^2}.$$

Minimizing with respect to the parameters κ_i and σ^2 (i.e., setting their derivatives to zero) results in the solution $\hat{\kappa}_i = \lambda_i$ for all i and $\hat{\sigma}^2 = \frac{\operatorname{tr}(\mathbf{C}_{22})}{n-d}$. In other words, $\hat{\mathbf{K}}_{11}$ has the same eigenvalues and eigenvectors as $\mathbf{C}_{11}$, that is,

$$\hat{\mathbf{K}}_{11} = \mathbf{C}_{11}.$$

This equation is satisfied if

$$\hat{\mathbf{B}} + \hat{\sigma}^2 \mathbf{V}\boldsymbol{\Gamma}_1^{-2}\mathbf{V}^T = \mathbf{V}\boldsymbol{\Gamma}_1^{-1}\mathbf{C}_{11}\boldsymbol{\Gamma}_1^{-1}\mathbf{V}^T$$

or

$$\hat{\mathbf{B}} = \mathbf{V}\boldsymbol{\Gamma}_1^{-1}(\mathbf{C}_{11} - \hat{\sigma}^2\mathbb{1})\boldsymbol{\Gamma}_1^{-1}\mathbf{V}^T$$

$\hat{\mathbf{B}}$ is positive semi-definite if and only if for all $v \in \mathbb{R}^d$

$$0 < \langle v, \hat{\mathbf{B}}v\rangle = \langle w, (\mathbf{C}_{11} - \hat{\sigma}^2\mathbb{1})w\rangle,$$

where $w = \boldsymbol{\Gamma}_1\mathbf{V}v$. Because $\mathbf{V}$ is unitary and $\boldsymbol{\Gamma}_1$ diagonal with strictly positive elements, $\langle v, \hat{\mathbf{B}}v\rangle > 0$ for all $v \in \mathbb{R}^d$ if and only if $\langle w, (\mathbf{C}_{11} - \hat{\sigma}^2\mathbb{1})w\rangle > 0$ for all $w \in \mathbb{R}^d$, or

$$0 < \min_{w\in\mathbb{R}^d} \frac{\langle w, \mathbf{C}_{11}w\rangle}{\langle w, w\rangle} - \hat{\sigma}^2 = \lambda_{\min}(\mathbf{C}_{11}) - \hat{\sigma}^2.$$

□

Eq. (S12) is a condition on the amount of variation in $\mathbf{Y}$ explained by the confounders $\mathbf{Z}$, with $\lambda_{\min}(\mathbf{C}_{11})$ being (proportional to) the minimum amount of variation explained by any of the dimensions spanned by the columns of $\mathbf{Z}$, and $\frac{1}{n-d}\operatorname{tr}(\mathbf{C}_{22})$ being the average amount of variation explained by the dimensions orthogonal to the columns of $\mathbf{Z}$. Failure of this condition simply means that there must be other, latent variables that explain more variation than the known ones, which is precisely what we are seeking to detect.

A useful special case of Theorem 1 occurs when the number of confounders equals one. In this case, we are seeking maximum-likelihood solutions for $\mathbf{K}$ of the form

$$\mathbf{K} = \beta^2 zz^T + \sigma^2\mathbb{1},$$

where $z \in \mathbb{R}^n$ is the confounding data vector. Let $\gamma^2 = \|z\|^2$ and $u = \frac{1}{\gamma}z$. Then $\mathbf{P}_z = uu^T$ is the projection matrix onto z, $\mathbf{C}_{11} = \langle u, \mathbf{C}u\rangle$, and $\operatorname{tr}(\mathbf{C}_{22}) = \operatorname{tr}((1-\mathbf{P}_z)\mathbf{C}) = \operatorname{tr}(\mathbf{C}) - \langle u, \mathbf{C}u\rangle$. By Theorem 1, we have

$$\begin{aligned}
\hat{\beta}^2 &= \frac{1}{\gamma^2}\left\{\langle u, \mathbf{C}u\rangle - \frac{\operatorname{tr}\big([1-\mathbf{P}_z]\mathbf{C}\big)}{n-1}\right\} \\
&= \frac{1}{\gamma^2}\left\{\frac{n}{n-1}\langle u, \mathbf{C}u\rangle - \frac{\operatorname{tr}(\mathbf{C})}{n-1}\right\} \\
\hat{\sigma}^2 &= \frac{\operatorname{tr}\big([1-\mathbf{P}_z]\mathbf{C}\big)}{n-1} = \frac{\operatorname{tr}(\mathbf{C}) - \langle u, \mathbf{C}u\rangle}{n-1},
\end{aligned} \tag{S16}$$

provided

$$\langle u, \mathbf{C}u\rangle > \frac{\operatorname{tr}(\mathbf{C})}{n}.$$

S5 Solution of the model without known covariates

Next, consider a model without known covariates, i.e. with posterior sample covariance matrix $\mathbf{K} = \mathbf{K}_X(\{\alpha_j, x_j\}) + \sigma^2 \mathbb{1}$, where

$$\mathbf{K}_X\big(\{\alpha_j, x_j\}\big) = \sum_{j=1}^{p} \alpha_j^2 x_j x_j^T.$$

This model is equivalent to probabilistic principal component analysis [3,4], and its maximum-likelihood solution is given by the first p eigenvectors or principal components with largest eigenvalues of $\mathbf{C}$. Here we present a more direct proof of this fact than what can be found in the literature.

Lemma 3. *Without loss of generality, we may assume that the latent variables have unit norm, are linearly independent, and are mutually orthogonal.*

Proof. If the latent variables do not have unit norm, define $c_j = \|x_j\|^{-1}$, $\alpha'_j = \alpha_j / c_j$ and $x'_j = c_j x_j$ for all j. It follows immediately that $\|x'_j\| = 1$ and

$$\mathbf{K}_X\big(\{\alpha_j, x_j\}\big) = \mathbf{K}_X\big(\{\alpha'_j, x'_j\}\big).$$

Next assume that the latent variables are not linearly independent, i.e. that $\text{rank}(\mathbf{K}_X) = r < p$. Because $\mathbf{K}_X$ is a symmetric matrix, we must have $\mathbf{K}_X = \sum_{l=1}^{r} t_l t_l^T$ for some set of linearly independent vectors $t_l \in \mathbb{R}^n$. Define $\alpha'_l = \|t_l\|$ and $x'_l = t_l / \|t_l\|$. Then x'_l has unit norm and

$$\mathbf{K}_X\big(\{\alpha'_l, x'_l\}\big) = \mathbf{K}_X\big(\{\alpha_j, x_j\}\big).$$

Finally, recall that

$$\mathbf{K}_X(\{\alpha_j, x_j\}) = \mathbf{X}\mathbf{A}\mathbf{X}^T = (\mathbf{X}\mathbf{A}^{\frac{1}{2}})(\mathbf{X}\mathbf{A}^{\frac{1}{2}})^T,$$

where $\mathbf{A} = \text{diag}(\alpha_1^2, \dots, \alpha_p^2)$. Because we may now assume that $\text{rank}(\mathbf{X}) = p$, and because $\alpha_j > 0$ for all j, the matrix $\mathbf{X}\mathbf{A}^{\frac{1}{2}}$ has singular value decomposition

$$\mathbf{X}\mathbf{A}^{\frac{1}{2}} = \mathbf{U}\Xi\mathbf{V}^T$$

with $\mathbf{U} \in \mathbb{R}^{n\times p}$, $\mathbf{U}^T\mathbf{U} = \mathbb{1}$, $\Xi \in \mathbb{R}^{p\times p}$ diagonal with diagonal elements $\Xi_{jj} = \xi_j > 0$, and $\mathbf{V} \in \mathbb{R}^{p\times p}$, $\mathbf{V}^T\mathbf{V} = \mathbf{V}\mathbf{V}^T = \mathbb{1}$. Hence

$$\mathbf{K}_X\big(\{\alpha_j, x_j\}\big) = \mathbf{U}\Xi^2\mathbf{U}^T = \sum_{j=1}^{p} \xi_j^2 u_j u_j^T = \mathbf{K}_X\big(\{\xi_j, u_j\}\big),$$

with u_j the orthonormal columns of $\mathbf{U}$, $\langle u_j, u'_j \rangle = (\mathbf{U}^T\mathbf{U})_{jj'} = \delta_{j,j'}$. □

We will also need the following simple result:

Lemma 4. *Let $\lambda_1 \geq \lambda_2 \geq \cdots \geq \lambda_n > 0$ be a decreasing sequence of positive numbers, and let $1 \leq p < n$. If there exists $j > p$ such that $\lambda_p > \lambda_j$, then*

$$\lambda_p > \frac{1}{n-p} \sum_{j=p+1}^{n} \lambda_j. \tag{S17}$$

Proof. Eq. (S17) follows from

$$\lambda_p - \frac{1}{n-p}\sum_{j=p+1}^{n}\lambda_j = \frac{1}{n-p}\sum_{j=p+1}^{n}(\lambda_p - \lambda_j) > 0,$$

because each term on the r.h.s. is non-negative, and at least one is strictly positive. □

Theorem 2. *Let* $\mathbf{C} \in \mathbb{R}^{n\times n}$ *be a positive definite matrix with eigenvalues* $\lambda_1 \geq \cdots \geq \lambda_n$ *and corresponding eigenvectors* $u_1, \dots, u_n$*, and let either* $p = n$ *or* $1 \leq p < n$ *such that there exists* $j > p$ *with* $\lambda_p > \lambda_j$*. Then the maximum-likelihood solution*

$$\hat{\mathbf{K}} = \operatorname*{argmin}_{\{\mathbf{K}:\, \mathbf{K}=\mathbf{X}\mathbf{A}\mathbf{X}^T+\sigma^2\mathbb{1}\}} \log\det\mathbf{K} + \operatorname{tr}\big(\mathbf{K}^{-1}\mathbf{C}\big),$$

is given by

$$\begin{aligned}\hat{x}_j &= u_j\\ \hat{\alpha}_j^2 &= \lambda_j - \hat{\sigma}^2\\ \hat{\sigma}^2 &= \frac{1}{n-p}\sum_{j=p+1}^{n}\lambda_j.\end{aligned}$$

Proof. By Lemma 3, we can assume that $\mathbf{X}$ has orthonormal columns, and hence there exist $\mathbf{V} \in \mathbb{R}^{n\times(n-p)}$ such that $\mathbf{Q} = (\mathbf{X}, \mathbf{V}) \in \mathbb{R}^{n\times n}$ is unitary, $\mathbf{Q}^T\mathbf{Q} = \mathbf{Q}\mathbf{Q}^T = \mathbb{1}$. Hence $\mathbf{K} = \mathbf{X}\mathbf{A}\mathbf{X}^T + \sigma^2\mathbb{1}$ has the spectral decomposition

$$\mathbf{K} = \begin{pmatrix}\mathbf{X} & \mathbf{V}\end{pmatrix}\begin{pmatrix}\mathbf{A}^2+\sigma^2\mathbb{1} & 0\\ 0 & \sigma^2\mathbb{1}\end{pmatrix}\begin{pmatrix}\mathbf{X}^T\\ \mathbf{V}^T\end{pmatrix},$$

and hence

$$\mathbf{K}^{-1} = \sum_{j=1}^{p}\frac{1}{\alpha_j^2+\sigma^2}x_j x_j^T + \frac{1}{\sigma^2}\sum_{l=1}^{n-p}v_l v_l^T,$$

where $v_l \in \mathbb{R}^n$ are the columns of $\mathbf{V}$.

Assume that the α_j^2 are ordered, $\alpha_1^2 \geq \cdots \geq \alpha_p^2$. Applying von Neumann's Lemma 2 gives

$$\begin{aligned}\mathcal{L} &= \log\det(\mathbf{K}) + \operatorname{tr}\big(\mathbf{K}^{-1}\mathbf{C}\big)\\ &\geq \sum_{j=1}^{p}\log(\alpha_j^2+\sigma^2) + (n-p)\log(\sigma^2) + \sum_{j=1}^{p}\frac{\lambda_j}{\alpha_j^2+\sigma^2} + \sum_{j=p+1}^{n}\frac{\lambda_j}{\sigma^2},\end{aligned} \tag{S18}$$

with equality if and only if

$$\begin{aligned}x_j &= u_j \text{ for } j = 1,\dots,p\\ v_l &= u_{p+l} \text{ for } l = 1,\dots,n-p\end{aligned}$$

Hence, independent of the values for α_j, the maximum-likelihood latent variables are the eigenvectors of $\mathbf{C}$ corresponding to the p largest eigenvalues. Minimizing eq, (S18) w.r.t. α_j^2

and σ^2 then gives

$$\alpha_j^2 = \lambda_j - \sigma^2$$
$$\sigma^2 = \frac{1}{n-p} \sum_{j=p+1}^{N} \lambda_j.$$

By Lemma 4, $\alpha_j^2 > 0$ for all j. □

Note that plugging the maximum-likelihood values in the likelihood function gives

$$\mathcal{L}_{\min} = \sum_{j=1}^{p} \log(\lambda_j) + (n-p) \log\Big(\frac{1}{n-p} \sum_{j=p+1}^{n} \lambda_j\Big) + n \tag{S19}$$

Either p can be set *a priori* small enough such that condition (S17) is satisfied, or else the value of p with smallest $\mathcal{L}_{\min}$ satisfying this condition can be found easily from eq. (S19).

Note also that in the models of [3,4], uniform prior variances are assumed ($\alpha_1^2 = \cdots = \alpha_p^2 = 1$), such that $\mathbf{X}$ is defined upto an arbitrary rotation, because $\mathbf{X}\mathbf{X}^T = (\mathbf{X}\mathbf{R})(\mathbf{X}\mathbf{R})^T$ for any rotation matrix $\mathbf{R}$. In our model, there is no such rotational freedom (if $\mathbf{A}$ is assumed to be diagonal), except if $\mathbf{C}$ has eigenvalues with multiplicities greater than one, when there is some freedom to choose the corresponding eigenvectors.

S6 Solution of the full model

S6.1 Orthogonality of known and hidden confounders

Lemma 5. *Without loss of generality, we may assume that the latent variables are orthogonal to the known confounders:*

$$\mathbf{X}^T\mathbf{Z} = \mathbf{Z}\mathbf{X}^T = 0. \tag{S20}$$

Proof. As in Section S4, let $\mathbf{P}_Z$ again be the projection matrix on the space spanned by the known covariates z_k (i.e. the columns of $\mathbf{Z}$). For any choice of latent variables x_j, we have

$$x_j = \mathbf{P}_Z x_j + (1 - \mathbf{P}_Z) x_j = \sum_{k=1}^{d} m_{kj} z_k + \tilde{x}_j,$$

for some matrix of linear coefficients $\mathbf{M} = (m_{kq}) \in \mathbb{R}^{d\times p}$, and with $\langle s_k, \tilde{x}_j \rangle = 0$ for all k. Or, in matrix notation

$$\mathbf{X} = \mathbf{Z}\mathbf{M} + \tilde{\mathbf{X}}, \quad \text{with} \quad \tilde{\mathbf{X}}^T\mathbf{Z} = \mathbf{Z}^T\tilde{\mathbf{X}} = 0$$

Plugging this in eq. (S2), results in

$$y_i = \mathbf{Z}\tilde{v}_i + \tilde{\mathbf{X}} w_i + \epsilon_i \tag{S21}$$

where $\tilde{v}_i = v_i + \mathbf{M}w_i$. Hence

$$\begin{bmatrix} \tilde{v}_i \\ w_i \\ \epsilon_i \end{bmatrix} = \begin{bmatrix} \mathbb{1} & M & 0 \\ 0 & \mathbb{1} & 0 \\ 0 & 0 & \mathbb{1} \end{bmatrix} \begin{bmatrix} v_i \\ w_i \\ \epsilon_i \end{bmatrix}$$

and hence, using Lemma 1, it follows that

$$p\left(\begin{bmatrix} \tilde{v}_i \\ w_i \\ \epsilon_i \end{bmatrix}\right) = \mathcal{N}\left(0, \begin{bmatrix} \mathbf{B} + \mathbf{M}\mathbf{D}^T + \mathbf{D}\mathbf{M}^T + \mathbf{M}\mathbf{A}\mathbf{M}^T & \mathbf{D} + \mathbf{A}\mathbf{M}^T & 0 \\ \mathbf{D}^T + \mathbf{M}\mathbf{A} & \mathbf{A} & 0 \\ 0 & 0 & \sigma^2\mathbb{1} \end{bmatrix}\right).$$

This is still of exactly the same form as eq. (S4). Hence model (S21) is identical to model (S2), but has hidden covariates orthogonal to the known covariates. □

Note that we can parameterize the model with hidden variables orthogonal to the known confounders, $\mathbf{Z}^T\mathbf{X} = 0$, but only if we allow the covariances of their effects on gene expression, $\mathrm{Cov}(v_i, w_i) = \mathbf{D}$, to be non-zero. Equivalently, we can parameterize the model such that the random effects of hidden variables are statistically independent of the effects of the known confounders, $\mathrm{Cov}(v_i, w_i) = 0$, but only if we allow the hidden variables to overlap with the known confounders, $\mathbf{Z}^T\mathbf{X} \neq 0$. Mathematically, the choice of orthogonal hidden factors will be much more convenient.

Note also that a transformation to orthogonal hidden factors always induces non-zero covariances among the known confounders via the term $\mathbf{M}\mathbf{A}\mathbf{M}^T$. Hence an important difficulty with the model where $\mathbf{B}$ is assumed to be diagonal, as used in [5], comes from the fact that non-orthogonal hidden variables are needed to model off-diagonal covariances between the known confounders. It is much more intuitive to model these directly by assuming a general covariance matrix.

S6.2 Restricted maximum-likelihood solution for the latent variables

Lemma 6. *Without loss of generality, we may assume that the latent variables have unit norm, are linearly independent, and are mutually orthogonal.*

Proof. The proof is identical to the proof of Lemma 3 – it is straightforward to verify that the transformation to orthonormal variables also do not change the form of the off-diagonal term $\mathbf{Z}\mathbf{D}\mathbf{X}^T$ in the covariance matrix $\mathbf{K}$, but merely lead to a reparameterization of the matrix $\mathbf{D}$. □

To solve the full model, we follow an approach similar to the standard restricted maximum-lilelihood method for linear mixed models [6,7]: we write the negative log-likelihood function $\mathcal{L} = \log\det(\mathbf{K}) + \mathrm{tr}(\mathbf{K}^{-1}\mathbf{C})$ as a sum

$$\mathcal{L} = \mathcal{L}_1 + \mathcal{L}_2, \tag{S22}$$

where $\mathcal{L}_2$ will be the log-likelihood restricted to the subspace orthogonal to the known confounders $\mathbf{Z}$. We will estimate the latent variables $\mathbf{X}$ and their effect covariances $\mathbf{A}$ by maximizing $\mathcal{L}_2$, and estimate the effect covariances $\mathbf{B}$ and $\mathbf{D}$ involving the known confounders

by maximizing $\mathcal{L}_1$. Solving for the latent variables on a restricted subspace is motivated by the observation that if $y \in \mathbb{R}^n$ is a sample from the model (S2), that is, $p(y) = \mathcal{N}(0, \mathbf{K})$, then

$$\mathbf{U}_2\mathbf{U}_2^T y = \mathbf{U}_2\mathbf{U}_2^T \mathbf{Z} v + \mathbf{U}_2\mathbf{U}_2^T \mathbf{X} w + \mathbf{U}_2\mathbf{U}_2^T \epsilon = \mathbf{X} w + \epsilon'.$$

In other words, restricted to the subspace orthogonal to $\mathbf{Z}$, the general model becomes a probablistic PCA model where all variation in the data is explained by the latent variables.

To obtain the decomposition (S22), we partition $y \in \mathbb{R}^n$ as $y = (y_1, y_2)^T$, where $y_1 = \mathbf{U}_1^T y \in \mathbb{R}^d$ and $y_2 = \mathbf{U}_2^T y \in \mathbb{R}^{n-d}$, and write

$$p(y) = p(y_1, y_2) = p(y_1 \mid y_2) p(y_2),$$

or

$$\log p(y) = \log p(y_1, y_2) = \log p(y_1 \mid y_2) + \log p(y_2).$$

Hence

$$\mathcal{L} = -\frac{2}{m}\sum_{i=1}^{m} \log p(y_i) = \underbrace{-\frac{2}{m}\sum_{i=1}^{m} \log p(y_{i1} \mid y_{i2})}_{\mathcal{L}_1} \underbrace{-\frac{2}{m}\sum_{i=1}^{m} \log p(y_{i2})}_{\mathcal{L}_2}$$

Using standard results for the marginal and conditional distributions of a multivariate Gaussian, we have

$$\begin{aligned} p(y_2) &= \mathcal{N}(0, \mathbf{K}_{22}) \\ p(y_1 \mid y_2) &= \mathcal{N}\big(\mathbf{K}_{12}\mathbf{K}_{22}^{-1} y_2, (\mathbf{K}_{11} - \mathbf{K}_{12}\mathbf{K}_{22}^{-1}\mathbf{K}_{21})\big), \end{aligned}$$

where we used the partitioned matrix notation of eq. (S8). In particular,

$$\begin{aligned} \mathcal{L}_2 &= \log\det(\mathbf{K}_{22}) + \frac{1}{m}\sum_{i=1}^{m} \langle \mathbf{U}_2^T y_i, \mathbf{K}_{22}^{-1}\mathbf{U}_2^T y_i \rangle \\ &= \log\det(\mathbf{K}_{22}) + \frac{1}{m}\sum_{i=1}^{m} \operatorname{tr}\big(\mathbf{K}_{22}^{-1}\mathbf{U}_2^T y_i y_i^T \mathbf{U}_2\big) \\ &= \log\det(\mathbf{K}_{22}) + \operatorname{tr}\big(\mathbf{K}_{22}^{-1}\mathbf{C}_{22}\big). \end{aligned}$$

Note that $\mathbf{K}_{22} = \mathbf{U}_2^T \mathbf{X}\mathbf{A}\mathbf{X}^T \mathbf{U}_2^T + \sigma^2 \mathbb{1}$, and hence $\mathcal{L}_2$ depends only on $\mathbf{X}$, $\mathbf{A}$ and σ^2. The restricted maximum likelihood solution for the latent variables follows immediately:

Theorem 3. *Let $\hat{\mathbf{X}} \in \mathbb{R}^{n\times p}$, $\hat{A} \in \mathbb{R}^{d\times d}$, and $\hat{\sigma}^2$ be the solution of*

$$\{\hat{\mathbf{X}}, \hat{\mathbf{A}}, \hat{\sigma}^2\} = \operatorname*{argmin}_{\mathbf{X},\mathbf{A},\sigma^2} \mathcal{L}_2(\mathbf{X}, \mathbf{A}, \sigma^2),$$

where the minimum is taken over all $\mathbf{X}$ with $\mathbf{X}^T\mathbf{Z} = 0$, and all positive semi-definite diagonal matrices $\hat{\mathbf{A}}$. If there exists $j > p$ such that $\lambda_p > \lambda_j$, then

$$\hat{\mathbf{X}} = \mathbf{U}_2 \mathbf{W}_p \tag{S23}$$

$$\hat{\mathbf{A}} = \operatorname{diag}(\lambda_1 - \hat{\sigma}^2, \dots, \lambda_p - \hat{\sigma}^2) \tag{S24}$$

$$\hat{\sigma}^2 = \frac{1}{n-d-p}\sum_{j=p+1}^{n-d} \lambda_j \tag{S25}$$

where $\lambda_1 \geq \lambda_2 \geq \cdots \geq \lambda_{n-d}$ are the sorted eigenvalues of $\mathbf{C}_{22}$ with corresponding eigenvectors $w_1, \dots, w_{n-d} \in \mathbb{R}^{n-d}$, and $\mathbf{W}_p = (w_1, \dots, w_p) \in \mathbb{R}^{(n-d)\times p}$ is the matrix with the first p eigenvectors of $\mathbf{C}_{22}$ as columns.

Proof. Defining $\tilde{\mathbf{X}} = \mathbf{U}_2^T\mathbf{X} \in \mathbb{R}^{(n-d)\times p}$, we have $\mathbf{K}_{22} = \tilde{\mathbf{X}}\mathbf{A}\tilde{\mathbf{X}}^T + \sigma^2\mathbb{1}$, and $\mathcal{L}_2$ becomes precisely the minus log-likelihood of the model without known covariates (Section S5), as a function of the latent variables $\tilde{\mathbf{X}}$ on the *reduced* $(n-d)$-dimensional space orthogonal to the known confounders $\mathbf{Z}$. Hence by Theorem 2,

$$\begin{aligned}\hat{\tilde{\mathbf{X}}} &= \mathbf{W}_p \\ \hat{\mathbf{A}} &= \operatorname{diag}(\lambda_1 - \sigma^2, \dots, \lambda_p - \sigma^2),\end{aligned}$$

where $\lambda_1 \geq \lambda_2 \geq \cdots \geq \lambda_{n-d}$ are the sorted eigenvalues of $\mathbf{C}_{22}$ and $\mathbf{W}_p \in \mathbb{R}^{(n-d)\times p}$ is the matrix having the corresponding first p eigenvectors as columns. Note that $\hat{\mathbf{A}}$ is positive semi-definite by Lemma 4 and the assumption that there exists $j > p$ such that $\lambda_p > \lambda_j$. It remains to 'pull-back' $\tilde{\mathbf{X}}$ to the original n-dimensional space, using the orthogonality condition (S20):

$$\hat{\mathbf{X}} = (\mathbf{U}_1\mathbf{U}_1^T + \mathbf{U}_2\mathbf{U}_2^T)\hat{\mathbf{X}} = \mathbf{U}_2\mathbf{U}_2^T\hat{\mathbf{X}} = \mathbf{U}_2\hat{\tilde{\mathbf{X}}} = \mathbf{U}_2\mathbf{W}_p.$$

This proves eqs. (S23) and (S24). □

S6.3 Solution for the variance parameters given the latent variables

With $\hat{\mathbf{X}}$, $\hat{\mathbf{A}}$ and $\hat{\sigma}^2$ determined by the minimization of $\mathcal{L}_2$ in Theorem 3, $\mathcal{L}_2(\hat{\mathbf{X}}, \hat{\mathbf{A}}, \hat{\sigma}^2)$ is constant in terms of the parameters $\mathbf{B}$ and $\mathbf{D}$ that remain to be optimized. Hence optimizing $\mathcal{L}_1$ with respect to these parameters is the same as optimizing the total negative log-likelihood $\mathcal{L}(\hat{\mathbf{X}}, \hat{\mathbf{A}}, \mathbf{B}, \mathbf{D}, \hat{\sigma}^2)$ w.r.t. $\mathbf{B}$ and $\mathbf{D}$. We have:

Theorem 4. *Let $\hat{\mathbf{B}} \in \mathbb{R}^{d\times d}$ and $\hat{D} \in \mathbb{R}^{d\times(n-d)}$ be the solution of*

$$\{\hat{\mathbf{B}}, \hat{\mathbf{D}}\} = \operatorname*{argmin}_{\mathbf{B},\mathbf{D}} \mathcal{L}_1(\hat{\mathbf{X}}, \hat{\mathbf{A}}, \mathbf{B}, \mathbf{D}, \hat{\sigma}^2) = \operatorname*{argmin}_{\mathbf{B},\mathbf{D}} \mathcal{L}(\hat{\mathbf{X}}, \hat{\mathbf{A}}, \mathbf{B}, \mathbf{D}, \hat{\sigma}^2),$$

subject to the constraint that $\mathbf{B}$ and $\mathbf{B} - \mathbf{D}\hat{\mathbf{A}}^{-1}\mathbf{D}^T$ are positive semi-definite. If

$$\lambda_{\min}(C_{11}) > \hat{\sigma}^2, \tag{S26}$$

then

$$\hat{\mathbf{B}} = \mathbf{V}\boldsymbol{\Gamma}_1^{-1}(\mathbf{C}_{11} - \hat{\sigma}^2\mathbb{1})\boldsymbol{\Gamma}_1^{-1}\mathbf{V}^T \tag{S27}$$

$$\hat{\mathbf{D}} = \mathbf{V}\boldsymbol{\Gamma}_1^{-1}\mathbf{C}_{12}\mathbf{W}_p \tag{S28}$$

where as before

$$\mathbf{Z} = \begin{pmatrix}\mathbf{U}_1 & \mathbf{U}_2\end{pmatrix}\begin{pmatrix}\boldsymbol{\Gamma}_1 \\ 0\end{pmatrix}\mathbf{V}^T$$

is the singular value decomposition of $\mathbf{Z}$, and $\mathbf{W}_p = (w_1, \dots, w_p) \in \mathbb{R}^{(n-d)\times p}$ is the matrix with the first p eigenvectors of $\mathbf{C}_{22}$ as columns.

Proof. Note that the conditions $\mathbf{B}$ and $\mathbf{B} - \mathbf{D}\hat{\mathbf{A}}^{-1}\mathbf{D}^T$ positive semi-definite are to ensure that the matrix $\begin{pmatrix} \mathbf{B} & \mathbf{D} \\ \mathbf{D}^T & \hat{\mathbf{A}} \end{pmatrix}$ is positive semi-definite. Next note that with $\hat{\mathbf{X}}^T$ known, the covariance matrix $\mathbf{K}$ can be written as

$$\mathbf{K} = \begin{pmatrix} \mathbf{Z} & \hat{\mathbf{X}} \end{pmatrix} \begin{pmatrix} \mathbf{B} & \mathbf{D} \\ \mathbf{D}^T & \hat{\mathbf{A}} \end{pmatrix} \begin{pmatrix} \mathbf{Z}^T \\ \hat{\mathbf{X}}^T \end{pmatrix} + \hat{\sigma}^2 \mathbb{1}$$

Hence the total log-likelihood is identical to the model with known covariates $\tilde{\mathbf{Z}} = \begin{pmatrix} \mathbf{Z} & \hat{\mathbf{X}} \end{pmatrix}$ and no latent variables (Section S4). The *unconstrained* maximizing solution (that is, where $\mathbf{A}$ and σ^2 are also optimized) for the model with known covariates $\tilde{\mathbf{Z}}$ is given by Theorem 1. Due to $\hat{\mathbf{X}}^T\mathbf{Z} = 0$ and the definition of $\hat{\mathbf{X}}$, the singular value decomposition of $\tilde{\mathbf{Z}}$ is given by

$$\tilde{\mathbf{Z}} = \begin{pmatrix} \mathbf{U}_1 & \hat{\mathbf{X}} & \mathbf{U}_3 \end{pmatrix} \begin{pmatrix} \mathbf{\Gamma}_1 & 0 \\ 0 & \mathbb{1} \\ 0 & 0 \end{pmatrix} \begin{pmatrix} \mathbf{V}^T & 0 \\ 0 & \mathbb{1} \end{pmatrix},$$

where the columns of $\mathbf{U}_3 \in \mathbb{R}^{n\times(n-d-p)}$ span the space orthogonal to the columns of $\tilde{\mathbf{Z}}$. Hence the unconstrained solution, can be written as (cf. eqs. (S14)–(S15))

$$\begin{pmatrix} \hat{\mathbf{B}} & \hat{\mathbf{D}} \\ \hat{\mathbf{D}}^T & \hat{\mathbf{A}}' \end{pmatrix} = \begin{pmatrix} \mathbf{V} & 0 \\ 0 & \mathbb{1} \end{pmatrix} \begin{pmatrix} \mathbf{\Gamma}_1^{-1} & 0 \\ 0 & \mathbb{1} \end{pmatrix} \begin{pmatrix} \mathbf{U}_1^T \\ \hat{\mathbf{X}}^T \end{pmatrix} (\mathbf{C} - \hat{\sigma}'^2 \mathbb{1}) \begin{pmatrix} \mathbf{U}_1 & \hat{\mathbf{X}} \end{pmatrix} \begin{pmatrix} \mathbf{\Gamma}_1^{-1} & 0 \\ 0 & \mathbb{1} \end{pmatrix} \begin{pmatrix} \mathbf{V}^T & 0 \\ 0 & \mathbb{1} \end{pmatrix}$$
$$\hat{\sigma}'^2 = \frac{\operatorname{tr}(\mathbf{U}_3^T \mathbf{C} \mathbf{U}_3)}{n-d-p}$$

First note that $\hat{\sigma}'^2 = \hat{\sigma}^2$, because we can write $\mathbf{U}_3 = \mathbf{U}_2 \mathbf{W}_{\sim p}$, where $\mathbf{W}_{\sim p} \in \mathbb{R}^{(n-d)\times(n-d-p)}$ is the matrix with the $n-d-p$ last eigenvectors of $\mathbf{C}_{22}$.

Working out the block matrix product results in:

$$\begin{aligned}
\hat{\mathbf{B}} &= \mathbf{V}\mathbf{\Gamma}_1^{-1}\mathbf{U}_1^T(\mathbf{C} - \hat{\sigma}^2\mathbb{1})\mathbf{U}_1\mathbf{\Gamma}_1^{-1}\mathbf{V}^T = \mathbf{V}\mathbf{\Gamma}_1^{-1}(\mathbf{C}_{11} - \hat{\sigma}^2\mathbb{1})\mathbf{\Gamma}_1^{-1}\mathbf{V}^T \\
\hat{\mathbf{D}} &= \mathbf{V}\mathbf{\Gamma}_1^{-1}\mathbf{U}_1^T\mathbf{C}\hat{\mathbf{X}} = \mathbf{V}\mathbf{\Gamma}_1^{-1}\mathbf{U}_1^T\mathbf{C}\mathbf{U}_2\mathbf{W}_p = \mathbf{V}\mathbf{\Gamma}_1^{-1}\mathbf{C}_{12}\mathbf{W}_p \\
\hat{\mathbf{A}}' &= \hat{\mathbf{X}}^T(\mathbf{C} - \hat{\sigma}^2\mathbb{1})\hat{\mathbf{X}} = \mathbf{W}_p^T\mathbf{U}_2^T(\mathbf{C} - \hat{\sigma}^2\mathbb{1})\mathbf{U}_2\mathbf{W}_p = \mathbf{W}_p^T(\mathbf{C}_{22} - \hat{\sigma}^2\mathbb{1})\mathbf{W}_p \\
&= \operatorname{diag}(\lambda_1 - \hat{\sigma}^2, \dots, \lambda_p - \hat{\sigma}^2)
\end{aligned}$$

Hence, also the estimate $\hat{\mathbf{A}}' = \hat{\mathbf{A}}$. Because the unconstrained optimization of $\mathcal{L}$ given $\hat{\mathbf{X}}$ results in the same estimate for $\mathbf{A}$ and σ^2 as the intial constrained optimization where these parameters were given, it follows that also the estimates of $\mathbf{B}$ and $\mathbf{D}$ must be the same:

$$\{\hat{\mathbf{B}}, \hat{\mathbf{D}}\} = \operatorname*{argmin}_{\mathbf{B},\mathbf{D}} \mathcal{L}(\mathbf{B}, \mathbf{D} \mid \hat{\mathbf{X}}, \hat{\mathbf{A}}, \hat{\sigma}^2) = \operatorname*{argmin}_{\mathbf{B},\mathbf{D}} \min_{\mathbf{A},\sigma^2} \mathcal{L}(\mathbf{A}, \mathbf{B}, \mathbf{D}, \sigma^2 \mid \hat{\mathbf{X}}).$$

□

S6.4 LVREML maximizes the variance explained

It is tempting to ask whether the combined solution from Theorems 3 and 4 optimizes the *total* likelihood among all possible p-dimensional sets of latent variables. To address this

problem, let $\mathbf{X} \in \mathbb{R}^{n\times p}$ be an arbitrary matrix of latent variables whose columns are normalized, mutually orthogonal and orthogonal to the columns of $\mathbf{Z}$, $\mathbf{X}^T\mathbf{X} = \mathbb{1}$ and $\mathbf{X}^T\mathbf{Z} = 0$. Because $\mathbf{U}_2$ is only defined upto a rotation, we can always choose

$$\mathbf{U}_2 = \begin{pmatrix}\mathbf{X} & \mathbf{Q}\end{pmatrix}$$

with $\mathbf{Q} \in \mathbb{R}^{n\times(n-d-p)}$ satisfying $\mathbf{Q}^T\mathbf{Q} = \mathbb{1}$, $\mathbf{Q}^T\mathbf{X} = 0$ and $\mathbf{Q}^T\mathbf{Z} = 0$. From the proof of Theorem 4 we immediately obtain:

Proposition 1. *Let* $\mathbf{A}(\mathbf{X}) \in \mathbb{R}^{p\times p}$, $\mathbf{B}(\mathbf{X}) \in \mathbb{R}^{d\times d}$, $\mathbf{D}(\mathbf{X}) \in \mathbb{R}^{d\times(n-d)}$ *and* $\sigma^2(\mathbf{X}) > 0$ *be the solution of*

$$\{\mathbf{A}(\mathbf{X}), \mathbf{B}(\mathbf{X}), \mathbf{D}(\mathbf{X}), \sigma^2(\mathbf{X})\} = \underset{\mathbf{A},\mathbf{B},\mathbf{D},\sigma^2}{\operatorname{argmin}} \mathcal{L}(\mathbf{A}, \mathbf{B}, \mathbf{D}, \sigma^2 \mid \mathbf{X}).$$

Then

$$\begin{aligned}
\mathbf{B}(\mathbf{X}) &= \mathbf{V}\mathbf{\Gamma}_1^{-1}(\mathbf{C}_{11} - \hat{\sigma}^2\mathbb{1})\mathbf{\Gamma}_1^{-1}\mathbf{V}^T \\
\mathbf{D}(\mathbf{X}) &= \mathbf{V}\mathbf{\Gamma}_1^{-1}\mathbf{U}_1^T\mathbf{C}\mathbf{X} \\
\mathbf{A}(\mathbf{X}) &= \mathbf{X}^T(\mathbf{C} - \hat{\sigma}^2\mathbb{1})\mathbf{X} \\
\sigma^2(\mathbf{X}) &= \frac{\operatorname{tr}(\mathbf{Q}^T\mathbf{C}\mathbf{Q})}{n-d-p}
\end{aligned}$$

□

Plugging these values into the negative log-likelihood function results in a function that depends only on $\mathbf{X}$:

Proposition 2. *Let* $\mathbf{X} \in \mathbb{R}^{n\times p}$ *be an arbitrary choice of latent variables with associated maximum-likelihood estimates for the covariance parameters given by Proposition 1. Then, upto an additive constant*

$$\mathcal{L}_{\mathbf{X}} = \log\det\Big(\mathbf{X}^T\big[\mathbf{C} - \mathbf{C}\mathbf{U}_1(\mathbf{U}_1^T\mathbf{C}\mathbf{U}_1)\mathbf{U}_1^T\mathbf{C}\big]\mathbf{X}\Big) + (n-d-p)\log\big(\hat{\sigma}^2(\mathbf{X})\big) \tag{S29}$$

Proof. Recall from Theorem 2 that the maximum-likelihood estimate for $\mathbf{K}$ given $\mathbf{X}$ and its associated maximum-likelihood parameters estimates is given by

$$\hat{\mathbf{K}}(\mathbf{X}) = \begin{pmatrix} \mathbf{U}_1^T\mathbf{C}\mathbf{U}_1 & \mathbf{U}_1^T\mathbf{C}\mathbf{X} & 0 \\ \mathbf{X}^T\mathbf{C}\mathbf{U}_1 & \mathbf{X}^T\mathbf{C}\mathbf{X} & 0 \\ 0 & 0 & \hat{\sigma}^2\mathbb{1} \end{pmatrix}$$

while the covariance matrix $\mathbf{C}$ can be written as

$$\mathbf{C} = \begin{pmatrix} \mathbf{U}_1^T\mathbf{C}\mathbf{U}_1 & \mathbf{U}_1^T\mathbf{C}\mathbf{X} & \mathbf{U}_1^T\mathbf{C}\mathbf{Q} \\ \mathbf{X}^T\mathbf{C}\mathbf{U}_1 & \mathbf{X}^T\mathbf{C}\mathbf{X} & \mathbf{X}^T\mathbf{C}\mathbf{Q} \\ \mathbf{Q}\mathbf{C}\mathbf{U}_1 & \mathbf{Q}\mathbf{C}\mathbf{X} & \mathbf{Q}^T\mathbf{C}\mathbf{Q} \end{pmatrix}$$

Hence

$$\begin{aligned}
\mathcal{L}_{\mathbf{X}} &= \mathcal{L}\big(\hat{\mathbf{K}}(\mathbf{X})\big) = \log\det\big(\hat{\mathbf{K}}(\mathbf{X})\big) + \mathrm{tr}\big(\hat{\mathbf{K}}(\mathbf{X})^{-1}\mathbf{C}\big) \\
&= \log\det\begin{pmatrix} \mathbf{U}_1^T\mathbf{C}\mathbf{U}_1 & \mathbf{U}_1^T\mathbf{C}\mathbf{X} \\ \mathbf{X}^T\mathbf{C}\mathbf{U}_1 & \mathbf{X}^T\mathbf{C}\mathbf{X} \end{pmatrix} + (n-d-p)\log(\hat{\sigma}^2) + (d+p) + \frac{\mathrm{tr}(\mathbf{Q}^T\mathbf{C}\mathbf{Q})}{\hat{\sigma}^2} \\
&= \log\det\begin{pmatrix} \mathbf{U}_1^T\mathbf{C}\mathbf{U}_1 & \mathbf{U}_1^T\mathbf{C}\mathbf{X} \\ \mathbf{X}^T\mathbf{C}\mathbf{U}_1 & \mathbf{X}^T\mathbf{C}\mathbf{X} \end{pmatrix} + (n-d-p)\log(\hat{\sigma}^2) + (d+p) + (n-d-p)
\end{aligned}$$

Using equation (S11) for the determinant of a partitioned matrix, we have

$$\begin{aligned}
\log\det\begin{pmatrix} \mathbf{U}_1^T\mathbf{C}\mathbf{U}_1 & \mathbf{U}_1^T\mathbf{C}\mathbf{X} \\ \mathbf{X}^T\mathbf{C}\mathbf{U}_1 & \mathbf{X}^T\mathbf{C}\mathbf{X} \end{pmatrix} &= \log\det(\mathbf{U}_1^T\mathbf{C}\mathbf{U}_1) + \log\det\big(\mathbf{X}^T\mathbf{C}\mathbf{X} - \mathbf{X}^T\mathbf{C}\mathbf{U}_1(\mathbf{U}_1^T\mathbf{C}\mathbf{U}_1)^{-1}\mathbf{U}_1\mathbf{C}\mathbf{X}\big) \\
&= \log\det(\mathbf{U}_1^T\mathbf{C}\mathbf{U}_1) + \log\det\Big(\mathbf{X}^T\left[\mathbf{C} - \mathbf{C}\mathbf{U}_1(\mathbf{U}_1^T\mathbf{C}\mathbf{U}_1)^{-1}\mathbf{U}_1^T\mathbf{C}\right]\mathbf{X}\Big).
\end{aligned}$$

Ignoring the constants $\log\det(\mathbf{U}_1^T\mathbf{C}\mathbf{U}_1)$ and n which do not depend on $\mathbf{X}$, we obtain eq. (S29). □

Due to the determinant term in eq. (S29), it is not clear whether the restricted maximum-likelihood solution $\hat{\mathbf{X}}$ of Theorem 3 (with its associated maximum-likelihood covariance parameters of Theorem 4) is the absolute minimizer of $\mathcal{L}_{\mathbf{X}}$,

$$\hat{\mathbf{X}} = \underset{\mathbf{X}\in\mathbb{R}^{n\times p}, \mathbf{X}^T\mathbf{X}=\mathbb{1}, \mathbf{X}^T\mathbf{Z}=0}{\mathrm{argmin}} \mathcal{L}_{\mathbf{X}} \quad ?$$

However, we do have the following result:

Theorem 5. *The restricted maximum-likelihood solution $\hat{\mathbf{X}}$ of Theorem 3 is the set of p latent variables that minimizes the residual variance among all choices of p latent variables,*

$$\hat{\mathbf{X}} = \underset{\mathbf{X}\in\mathbb{R}^{n\times p}, \mathbf{X}^T\mathbf{X}=\mathbb{1}, \mathbf{X}^T\mathbf{Z}=0}{\mathrm{argmin}} \sigma^2(\mathbf{X})$$

Proof. By Proposition 1 and the arguments leading up to it, we can write

$$\mathrm{tr}(\mathbf{C}_{22}) = \mathrm{tr}(\mathbf{X}^T\mathbf{C}\mathbf{X}) + \mathrm{tr}(\mathbf{Q}^T\mathbf{C}\mathbf{Q}^T) = \mathrm{tr}\Big((\mathbf{U}_2^T\mathbf{X})^T\mathbf{C}_{22}(\mathbf{U}_2^T\mathbf{X})\Big) + \mathrm{tr}\Big((\mathbf{U}_2^T\mathbf{Q})^T\mathbf{C}_{22}(\mathbf{U}_2^T\mathbf{Q})\Big),$$

where as before $\mathbf{C}_{22} = \mathbf{U}_2^T\mathbf{C}\mathbf{U}_2$ is the restriction of $\mathbf{C}$ to the $(n-d)$-dimensional subspace orthogonal to the d known covariates, and the columns of $\mathbf{U}_2^T\mathbf{X}$ and $\mathbf{U}_2^T\mathbf{Q}$ span mutually orthogonal subspaces within this $(n-d)$-dimensional space. Hence $(n-d-p)\sigma^2(\mathbf{X}) = \mathrm{tr}(\mathbf{Q}^T\mathbf{C}\mathbf{Q}^T)$ is the trace of $\mathbf{C}_{22}$ over the residual $(n-d-p)$-dimensional space orthogonal to the latent variables, within the subspace orthogonal to the d known covariates. By the Courant-Fisher min-max theorem for eigenvalues [2], the $(n-d-p)$-dimensional subspace of $\mathbb{R}^{n-d}$ with *smallest* trace is the subspace spanned by the eigenvectors of $\mathbf{C}_{22}$ corresponding to its $(n-d-p)$ smallest eigenvalues. By Theorem 3, this is exactly the subspace obtained by choosing $\mathbf{X}$ equal to the restricted maximum-likelihood solution $\hat{\mathbf{X}}$. □

S7 Selecting covariates and the latent dimension

Two practical problems remain: how to choose the latent variable dimension parameter p and which known covariates to include?

To choose p, we will use the following result:

Lemma 7.

$$\operatorname{tr}(\mathbf{C}) = \operatorname{tr}(\hat{\mathbf{K}}) = \operatorname{tr}(\mathbf{Z}\hat{\mathbf{B}}\mathbf{Z}^T) + \operatorname{tr}(\hat{\mathbf{X}}\hat{\mathbf{A}}\hat{\mathbf{X}}^T) + n\hat{\sigma}^2$$

Proof. Use Theorem 4 to compute

$$\begin{aligned}
\operatorname{tr}(\mathbf{Z}\hat{\mathbf{B}}\mathbf{Z}) &= \operatorname{tr}\Big(\mathbf{U}_1\mathbf{\Gamma}_1\mathbf{V}^T\big[\mathbf{V}\mathbf{\Gamma}_1^{-1}(\mathbf{C}_{11} - \hat{\sigma}^2\mathbb{1})\mathbf{\Gamma}_1^{-1}\mathbf{V}^T\big]\mathbf{V}\mathbf{\Gamma}_1\mathbf{U}_1^T\Big) \\
&= \operatorname{tr}\big(\mathbf{U}_1\mathbf{C}_{11}\mathbf{U}_1^T\big) - \hat{\sigma}^2\operatorname{tr}\big(\mathbf{U}_1\mathbf{U}_1^T\big) \\
&= \operatorname{tr}(\mathbf{C}_{11}) - d\hat{\sigma}^2,
\end{aligned}$$

where the last step uses the cyclical property of the trace and the fact that $\mathbf{U}_1^T\mathbf{U}_1 = \mathbb{1}_d$. Likewise, we have

$$\begin{aligned}
\operatorname{tr}\big(\hat{\mathbf{X}}\hat{\mathbf{A}}\hat{\mathbf{X}}\big) &= \operatorname{tr}\Big(\mathbf{U}_2\mathbf{W}_p\operatorname{diag}(\lambda_1,\dots,\lambda_p)\mathbf{W}_p^T\mathbf{U}_2^T\Big) - \hat{\sigma}^2\operatorname{tr}\big(\mathbf{U}_2\mathbf{W}_p\mathbf{W}_p^T\mathbf{U}_2^T\big) \\
&= \sum_{j=1}^{p}\lambda_j - p\hat{\sigma}^2 \\
&= \sum_{j=1}^{n-d}\lambda_j - (n-d)\hat{\sigma}^2 \\
&= \operatorname{tr}(\mathbf{C}_{22}) - (n-d)\hat{\sigma}^2.
\end{aligned}$$

Hence

$$\operatorname{tr}(\hat{\mathbf{K}}) = \operatorname{tr}(\mathbf{Z}\hat{\mathbf{B}}\mathbf{Z}) + \operatorname{tr}(\hat{\mathbf{X}}\hat{\mathbf{A}}\hat{\mathbf{X}}) + n\hat{\sigma}^2 = \operatorname{tr}(\mathbf{C}_{11}) + \operatorname{tr}(\mathbf{C}_{22}) = \operatorname{tr}(\mathbf{C})$$

□

Because $\mathbf{C} = (\mathbf{Y}\mathbf{Y}^T)/m$, the eigenvalues of $\mathbf{C}$ are (proportional to) the squared singular values of the expression data $\mathbf{Y}$. Hence $\operatorname{tr}(\mathbf{Z}\hat{\mathbf{B}}\mathbf{Z})/\operatorname{tr}(\mathbf{C})$ is the proportion of variation in $\mathbf{Y}$ explained by the known covariates, $\operatorname{tr}(\hat{\mathbf{X}}\hat{\mathbf{A}}\hat{\mathbf{X}})/\operatorname{tr}(\mathbf{C})$ the proportion of variation explained by the latent variables, and $n\hat{\sigma}^2/\operatorname{tr}(\mathbf{C})$ is the residual variance.

Our method for determining the number of latent variables lets the user decide *a priori* the minimum amount of variation ρ in the data that should be explained by the known and latent confounders. It follows that given ρ, a "target" value for σ^2 is

$$\sigma^2(\rho) = \min\Big\{\frac{(1-\rho)\operatorname{tr}(\mathbf{C})}{n}, \lambda_{\min}(\mathbf{C}_{11})\Big\},$$

where the minimum with $\lambda_{\min}(\mathbf{C}_{11})$ is taken to ensure that of condition (S26) remains valid. Because the eigenvalues $\lambda_1, \dots, \lambda_{n-d}$ are sorted, the function

$$f(p) = \frac{1}{n-d-p} \sum_{j=p+1}^{n-d} \lambda_j$$

increases with decreasing p. Hence given ρ, we define $\hat{p}$ as

$$\hat{p} = \min\{p \colon 0 \leq p < n-d, \lambda_p > \lambda_{n-d}, f(p) < \sigma^2(\rho)\},$$

that is, we choose $\hat{p}$ to be the *smallest* number of latent variables that explain *at least* a propori-tion of variation ρ of $\mathbf{Y}$, while guaranteeing that the conditions for *all* mathematical results derived in this document are valid.

Note that unless all eigenvalues of $\mathbf{C}_{22}$ are identical, $\hat{p}$ always exists. Once the desired number of latent variables $\hat{p}$ is defined, the latent factors $\hat{\mathbf{X}}$, the variance parameters $\hat{\mathbf{A}}$, and the residual variance estimate $\hat{\sigma}^2$ (which will be the largest possible value less than or equal to the target value $\sigma^2(\rho)$) are determined by Theorem 3. Once those are determined, the remaining covariance parameters $\hat{\mathbf{B}}$ and $\hat{\mathbf{D}}$ are determined by Theorem 4.

A second practical problem occurs when the rank of $\mathbf{Z}$ exceeds the number of samples, such that any subset of n linearly independent covariates explains *all* of the variation in $\mathbf{Y}$. To select a more relevant subset of covariates, we rapidly screen all candidate covariates using the model with a single known covariate (Section S4) to compute the variance $\hat{\beta}^2$ explained by that covariate alone (eq. (S16)). We then keep only those covariates for which $\hat{\beta}^2 \geq \theta \operatorname{tr}(\mathbf{C})$, where $\theta > 0$ is the second free parameter of the method, namely the minimum amount of variation explained by a known covariate on its own. The selected covariates are ranked according to their value of $\hat{\beta}^2$, and a linearly independent subset is generated, starting from the covariates with highest $\hat{\beta}^2$.

S8 Downstream analyses

The inferred maximum-likelihood hidden factors $\hat{\mathbf{X}}$ and sample covariance matrix $\hat{\mathbf{K}}$ are typically used to create a dataset of residuals corrected for spurious sample correlations, to increase the power for detecting eQTLs, or as data-derived endophenotypes [5,8]. We briefly review these tasks and how they compare between LVREML and PANAMA hidden factors.

S8.1 Correcting data for spurious sample correlations

To remove spurious correlations due to the known and latent variance components from the expression data $\mathbf{Y} \in \mathbb{R}^{n\times m}$ (see Section S2), the residuals $\hat{\mathbf{y}}_i \in \mathbb{R}^n$ for gene i with original data $\mathbf{y}_i$ (a column of $\mathbf{Y}$) are contructed as

$$\hat{\mathbf{y}}_i = \hat{\mathbf{K}} \left(\sigma^2_{c,i}\hat{\mathbf{K}} + \sigma^2_{e,i}\mathbb{1}\right)^{-1} \hat{\mathbf{y}}_i$$

where the variance parameters $\sigma^2_{c,i}$ and $\sigma^2_{e,i}$ are fit separately for each gene i [5]. Hence two solutions for the latent factors that give rise to the same $\hat{\mathbf{K}}$ (as observed in Section 2.3 for LVREML and PANAMA) will result in the same residuals.

S8.2 Adjusting for known and latent covariates in eQTL association analyses

Two approaches for mapping eQTLs are commonly used in this context. The first approach tests for an association between SNP $\mathbf{s}_j$ and gene $\mathbf{y}_i$ using a mixed model, where the SNP is treated as a fixed effect, constructing likelihood ratio statistics as

$$\text{LOD}_{i,j} = \log \frac{\mathcal{N}(\mathbf{y}_i \mid \theta \mathbf{s}_j, \sigma_{c,i}^2 \hat{\mathbf{K}} + \sigma_{e,i}^2 \mathbb{1})}{\mathcal{N}(\mathbf{y}_i \mid 0, \sigma_{c,i}^2 \hat{\mathbf{K}} + \sigma_{e,i}^2 \mathbb{1})},$$

where the variance parameters $\sigma_{c,i}^2$ and $\sigma_{e,i}^2$ are fit separately for each gene i [5]. Hence for latent factor solutions that give rise to the same $\hat{\mathbf{K}}$ the association analyses will again be identical.

The second approach performs a linear regression of a gene's expression data, typically using the corrected data $\hat{\mathbf{y}}_i$, on the SNP genotypes $\mathbf{s}_j$, using the known and inferred factors as covariates [8], that is, a linear model is fit where

$$\hat{\mathbf{y}}_i = \beta_{i,j} \mathbf{s}_j + \mathbf{Z}\mathbf{a}_i + \hat{\mathbf{X}}\mathbf{b}_i + \epsilon_i \tag{S30}$$

where $\mathbf{Z}$ and $\hat{\mathbf{X}}$ are the matrices of known and estimated latent factors, respectively, and $\mathbf{a}_i \in \mathbb{R}^d$ and $\mathbf{b}_i \in \mathbb{R}^p$ are their respective regression coefficients.

Since maximum-likelihood solutions for the hidden factors by LVREML and PANAMA differ by a linear combination with the known factors $\mathbf{Z}$ that transforms models with hidden factors orthogonal to $\mathbf{Z}$ to equivalent models with hidden factors overlapping with $\mathbf{Z}$, and vice versa (see Section S6.1), it is clear that the same linear transformation will also result in equivalent linear association models in eq. (S30). Hence this type of analysis will also be equivalent between the hidden factors inferred by both approaches.

S8.3 Mapping the genetic architecture of latent variables

Inferred latent variables are sometimes treated as endophenotypes whose genetic architecture is of interest. In this case SNPs are identified that are strongly associated with the latent variables. Different solutions for the latent variables will then clearly result in different sets of significantly associated SNPs.

Using the maximum-likelihood LVREML inferred latent variables that are orthogonal to known confounders is advantageous in this context, because

- The LVREML latent variables are *uniquely defined*. All other solutions that give rise to the same covariance matrix estimate $\hat{\mathbf{K}}$ can be written as a linear combination of the known covariates and the LVREML covariates (see Section S6.1).
- When interpreting associated SNPs, there is no risk of attributing biological meaning to a latent variable that is due to the signal coming from the overlapping known covariates.

To remove the dependence of genetic association analyses on the choice of equivalent sets of latent variables, we recommend performing a multi-trait GWAS on the joint set of known

and latent confounders. If the standard multivariate association test based on canonical correlation analysis [9] is used, results will again be identical between equivalent choices of latent variables, because together with the known confounders they all span the same linear subspace.

Bibliography

[1] Theodore Wilbur Anderson and Ingram Olkin. Maximum-likelihood estimation of the parameters of a multivariate normal distribution. *Linear algebra and its applications*, 70:147–171, 1985.

[2] R A Horn and C R Johnson. *Matrix analysis*. Cambridge University Press, 1985.

[3] Michael E Tipping and Christopher M Bishop. Probabilistic principal component analysis. *Journal of the Royal Statistical Society: Series B (Statistical Methodology)*, 61(3):611–622, 1999.

[4] Neil Lawrence. Probabilistic non-linear principal component analysis with gaussian process latent variable models. *Journal of Machine Learning Research*, 6(Nov):1783–1816, 2005.

[5] Nicoló Fusi, Oliver Stegle, and Neil D Lawrence. Joint modelling of confounding factors and prominent genetic regulators provides increased accuracy in genetical genomics studies. *PLoS Computational Biology*, 8(1):e1002330, 2012.

[6] H Desmond Patterson and Robin Thompson. Recovery of inter-block information when block sizes are unequal. *Biometrika*, 58(3):545–554, 1971.

[7] FN Gumedze and TT Dunne. Parameter estimation and inference in the linear mixed model. *Linear Algebra and its Applications*, 435(8):1920–1944, 2011.

[8] Oliver Stegle, Leopold Parts, Matias Piipari, John Winn, and Richard Durbin. Using probabilistic estimation of expression residuals (peer) to obtain increased power and interpretability of gene expression analyses. *Nature Protocols*, 7(3):500–507, 2012.

[9] Manuel AR Ferreira and Shaun M Purcell. A multivariate test of association. *Bioinformatics*, 25(1):132–133, 2009.
